# Assessing saliva microbiome collection and processing methods

**DOI:** 10.1038/s41522-021-00254-z

**Published:** 2021-11-18

**Authors:** Abigail J. S. Armstrong, Veenat Parmar, Martin J. Blaser

**Affiliations:** Center for Advanced Biotechnology and Medicine, Rutgers University, Piscataway, NJ 08854 USA

**Keywords:** Metagenomics, Next-generation sequencing, Microbiome

## Abstract

The oral microbiome has been connected with lung health and may be of significance in the progression of SARS-CoV-2 infection. Saliva-based SARS-CoV-2 tests provide the opportunity to leverage stored samples for assessing the oral microbiome. However, these collection kits have not been tested for their accuracy in measuring the oral microbiome. Saliva is highly enriched with human DNA and reducing it prior to shotgun sequencing may increase the depth of bacterial reads. We examined both the effect of saliva collection method and sequence processing on measurement of microbiome depth and diversity by *16S rRNA gene* amplicon and shotgun metagenomics. We collected 56 samples from 22 subjects. Each subject provided saliva samples with and without preservative, and a subset provided a second set of samples the following day. *16S rRNA* gene (V4) sequencing was performed on all samples, and shotgun metagenomics was performed on a subset of samples collected with preservative with and without human DNA depletion before sequencing. We observed that the beta diversity distances within subjects over time was smaller than between unrelated subjects, and distances within subjects were smaller in samples collected with preservative. Samples collected with preservative had higher alpha diversity measuring both richness and evenness. Human DNA depletion before extraction and shotgun sequencing yielded higher total and relative reads mapping to bacterial sequences. We conclude that collecting saliva with preservative may provide more consistent measures of the oral microbiome and depleting human DNA increases yield of bacterial sequences.

## Introduction

The oral microbiome has high biomass second only to the gut^1^, and has been connected with both local^2,3^ and systemic illnesses^2,4^. Composition of the oral microbiome also may be a proxy for the microbiomes in other niches, including that of the gut^5^. The oral microbiome has a strong connection with the lung microbiome as oral microbes are enriched in pulmonary fluids^6–8^, and their presence in the lung has also been associated with inflammation^7^. As such, the oral microbiome not only provides an easily accessible proxy of lung microbes but also a potential indicator of lung health. The oral microbiome has shown promise as a diagnostic tool for SARS-CoV-2 infection detection and a predictor of disease severity^9–11^. Further, the oral microbiome may be an accessible niche for potential targeted therapeutics to reduce disease burden in the COVID-19 pandemic^11–14^.

An important advance in pandemic control has been the development of saliva-based assays to test for SARS-CoV-2 infection^15,16^. Saliva-based SARS-CoV-2 tests provide the opportunity to leverage archived samples for measuring the oral microbiome. However, the saliva collection kits designed to preserve SARS-CoV-2 RNA have not been developed nor validated for their ability to preserve an accurate representation of the oral microbiome. While several collection methods for the oral microbiome have been shown to have low impact on microbiome measurement^17,18^, the use of these kits for “off-label” usage remains untested. This testing is important if the samples archived are to be studied for their oral microbiome features, since many samples were obtained with a preservative. Furthermore, since saliva has high concentrations of human DNA, microbiome measurement using shotgun metagenomic approaches is expensive^19^. While methods to deplete human DNA have been tested^19^, the use of depletion on preserved samples has not.

In this study, our goal was to examine both the effect of saliva collection method and sequence processing on measurement of microbiome depth and diversity using both *16S rRNA* genes and shotgun metagenomics. We hypothesized that saliva collected with preservative will not have substantial differences in the microbiome composition as measured by *16S rRNA* gene sequencing compared to saliva collected without preservative. We further hypothesized that depleting human DNA preceding DNA extraction of saliva will increase the depth of the bacterial reads in shotgun metagenomic sequencing but will not have significant impact on the microbiome composition. To this end, we performed *16S rRNA* gene (V4) sequencing on saliva collected with and without preservative as well as shotgun metagenomics on a subset of the samples with and without human DNA depletion before sequencing.

## Results and discussion

### Study design

**Fig. 1:**
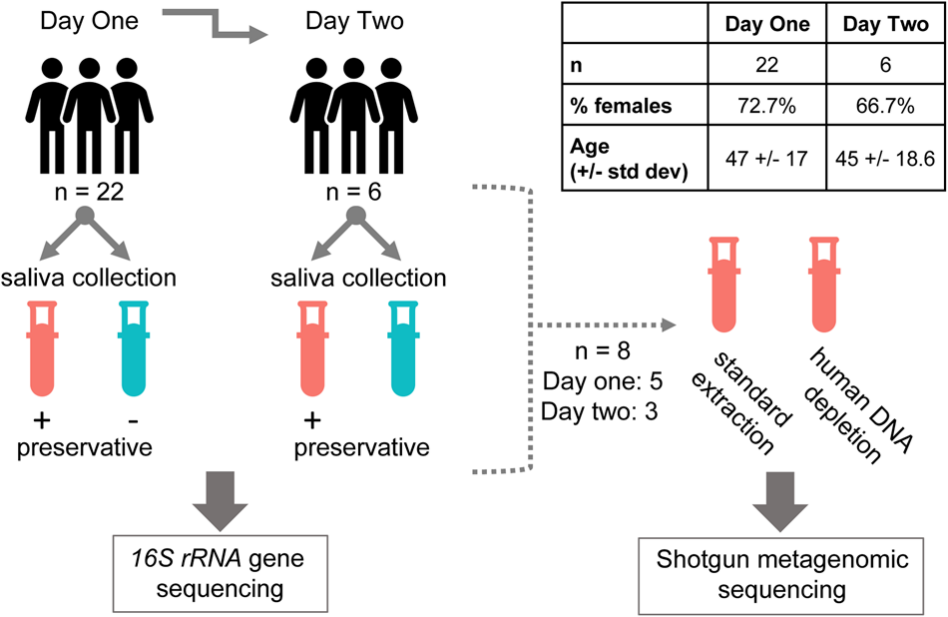
Saliva collection study design. Each subject provided two saliva samples at the same time, one collected alone and the other collected using the Spectrum sDNA-1000 kit which includes a sample preservative. The order in which samples were collected was randomized between subjects. The six subjects who provided two longitudinal samples provided the second sample the day following their initial saliva sample, using the same collection protocol with samples collected in the same order as on the previous day. For metagenomic studies, we assessed the effects of a protocol to deplete human DNA, using only samples in which the original preservative was used (*n* = 14 samples). We compared results in which the depletion technique was used or not; for six of the samples, DNA quantity was insufficient for metagenomics, so eight were studied in all.

We collected 56 samples from 22 subjects (Table 1). Each subject provided two saliva samples with and without preservative; six subjects provided a second set of samples the following day. *16S rRNA* gene (V4) sequencing was performed on all of the samples, and shotgun metagenomics was performed on eight of the samples collected with preservative with and without human DNA depletion before sequencing (Fig. 1).

**Table 1:** Cohort description.

	Day 1	Day 2
*n*	22	6
% Female	72.7	66.7
Median age	48	44.5
Smoking (never/past)	15/7	3/3
Vaping (never/past)	21/1	6/0
Probiotic usage (yes/no)	17/5	3/3

### DNA extraction and sequencing differences

**Fig. 2:**
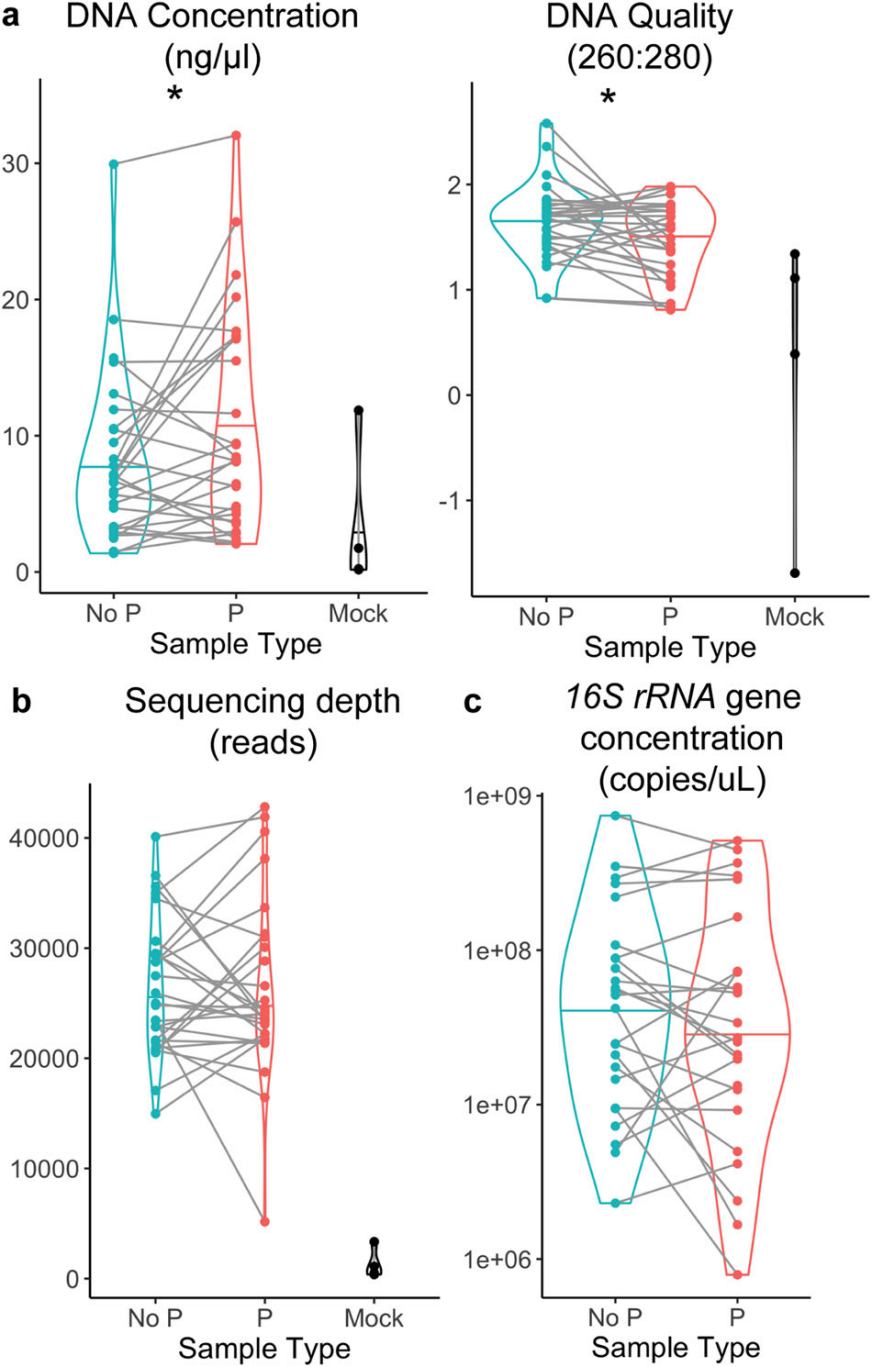
DNA extraction and sequencing, according to saliva collection method. The figure compares samples in preservative (P), or not (No P), or mock samples. **a** DNA quantity and quality post-extraction; *p < 0.05, linear mixed effect model. **b** Sequencing depth after processing in QIIME2; not significant, linear mixed effect model. **c** *16S rRNA* gene concentration in the two sample types; not significant, linear mixed effect model. Center line on plots indicates median.

DNA concentrations were higher in the samples collected with preservative compared to no preservative with 57.1% of samples increasing in concentration (mean ± SD: 10.51 + 7.96 vs 8.14 + 6.19, *p* = 0.04, paired *t*-test; Fig. 2a). The quality, as measured by 260:280 ratio, although slightly lower was still in an acceptable range with 32.1% of samples increasing in quality when collected with preservative (mean ± SD: 1.48 + 0.35 vs 1.65 + 0.36, *p* = 0.03, paired *t*-test; Fig. 2a). There was no difference in *16S rRNA* gene sequencing read depth between the two groups (mean ± SD: 26143 + 8065 vs 26199 + 6198, *p* = 0.97, paired *t*-test; Fig. 2b), suggesting that any difference in extraction efficiency did not impact *16S rRNA* gene PCR amplification or sequencing capability. Additionally, there was no significant difference in the amount of bacterial DNA, as measured by *16S rRNA* gene concentration, between the two sample collection methods. These result are consistent with prior work examining collection method on DNA yield of saliva samples^17^ and suggest that the preservative does not alter the ability to accurately collect and sequence microbial DNA compared to un-preserved saliva samples.

### 16*S rRNA* gene sequencing community metrics

**Fig. 3:**
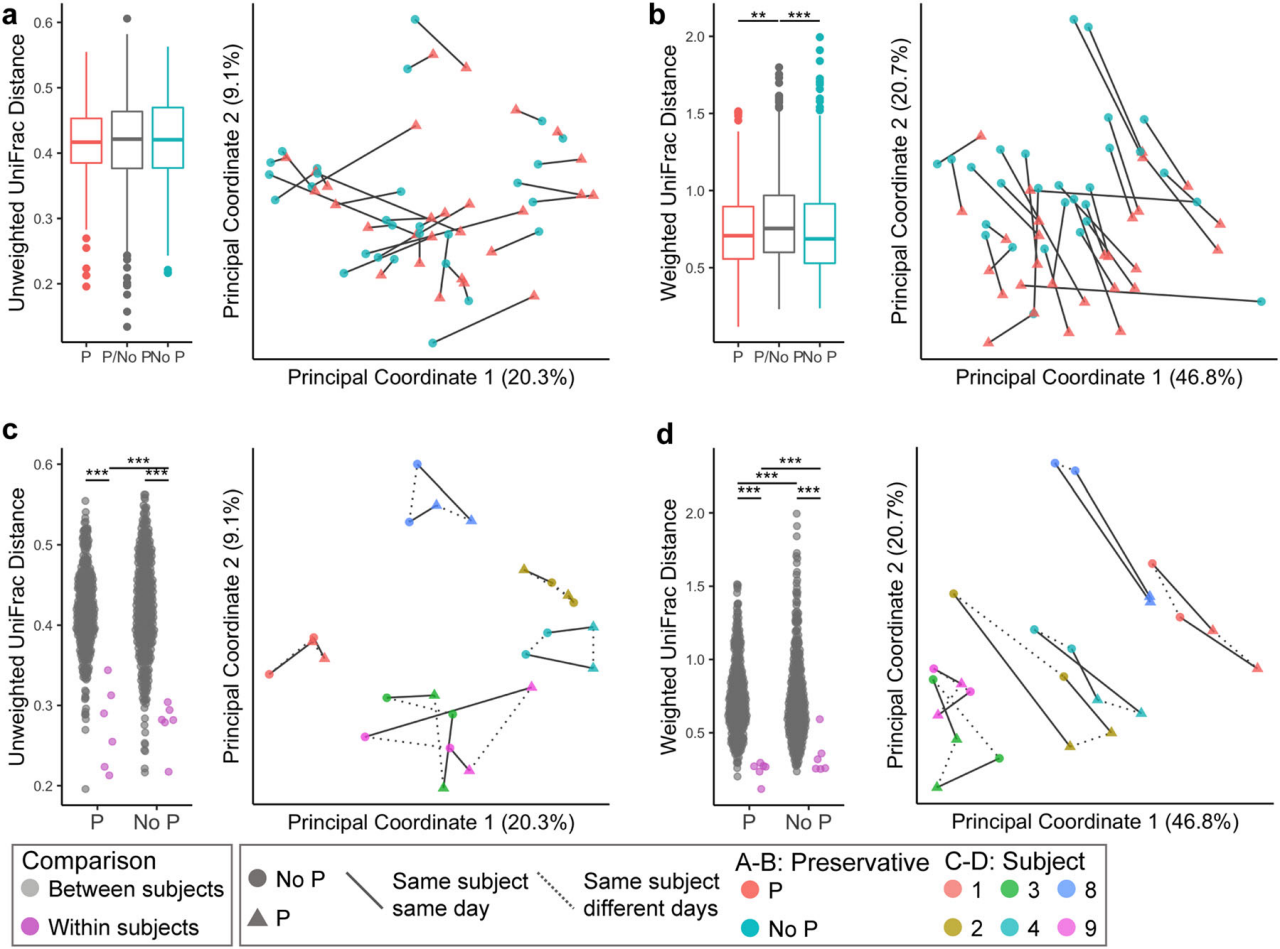
Phylogenetic beta diversity for *16S rRNA* gene analyses of saliva samples by collection method and across time. Top panels: unweighted (**a**) and weighted (**b**) UniFrac distance of all samples according to the collection method. Left panels: median (and IQR) distances in within-sample comparisons: preservative samples only (P), non-preservative samples (No P), and across sample sample collection methods—preservative vs non-preservative samples (P/No P). Right panels: PCoA plots of all samples by the sample collection method. Bottom panels: unweighted (**c**) and weighted (**d**) UniFrac distances of the paired specimens from six subjects sampled on two consecutive days and according to collection method. Left panels: distance between unrelated subjects (gray) or within an individual across days (pink) separated by collection method: preservative samples (P) and non-preservative samples (no P). Right panels: PcoA plot of all samples with only six multi-day subjects visualized. For all left panels, pairwise Wilcox test with FDR correction **q < 0.01, ***q < 0.001. Lines connect specimens collected from the same subject on the same day (solid) or different days (dotted).

First, we asked whether the population structure in the samples was affected by collection with preservative using phylogeny-based beta diversity metrics. There was significant clustering by the collection method in the weighted but not the unweighted UniFrac (Fig. 3a, b; PERMANOVA test *p* = 0.01 and *p* = 0.27 respectively). These results provide evidence that the preservative does not have a significant effect on the overall composition in terms of presence/absence of microbes in the community (unweighted UniFrac), but does affect the abundance of some community members (weighted UniFrac). As expected, distances within subjects over time were smaller than the distances between unrelated subjects regardless of collection method (Fig. 3c, d). However, the distances within samples from subjects collected with preservative were smaller than those collected without. Similar patterns were also seen when the Jaccard and Bray–Curtis beta diversity metrics were employed (Supplementary Fig. 1). In total, these findings show substantial similarity in the community composition between collection methods and suggest that samples collected with preservative may provide more stable sampling of the saliva microbiome over time.

**Fig. 4:**
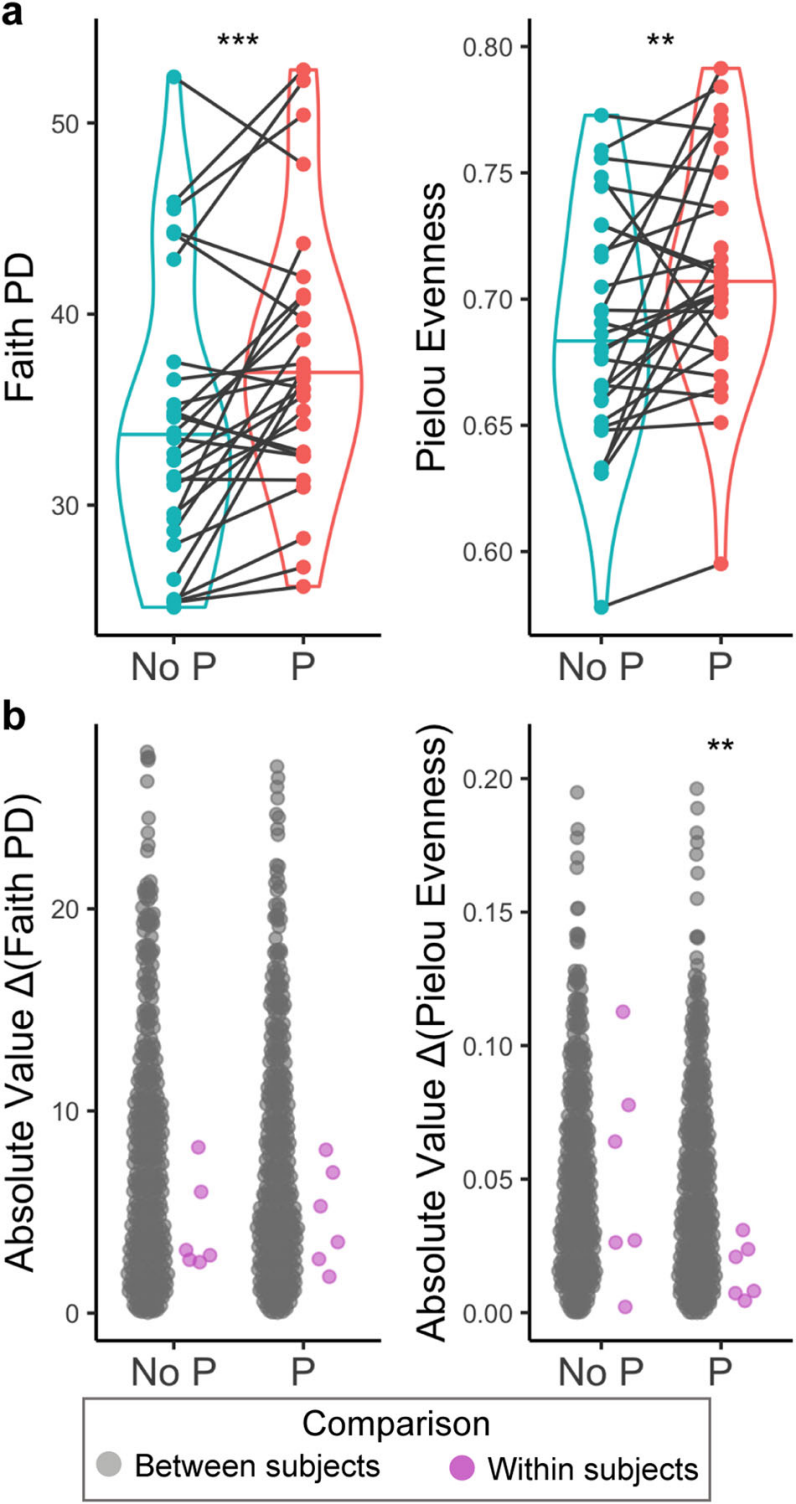
Alpha diversity of samples, by collection method and over time. **a** Alpha diversity measures based on Faith PD and Pielou evenness by sample collection with preservative (P) or not (No P). Lines connecting points indicate sample pairs. **p < 0.01, ***p < 0.001; linear mixed effects model. Center line on plots indicates median. **b** Absolute value of the differences in alpha diversity between all unrelated subjects (pink circles), and longitudinal samples within the same subject (gray circles). *p < 0.05, **p < 0.01**;** Kruskal–Wallis test.

Samples collected with preservative had higher alpha diversity measurements in analyses evaluating both richness and evenness (Fig. 4a and Supplementary Fig. 2A). One interpretation is that the use of the preservative may be preserving DNA from more species in the course of sample transit/processing, as evidenced by the higher richness, as well as preventing potential blooms of species growing during the same process, as evidenced by higher evenness. In any event, the use of preservative did not diminish the species richness detected in the samples. We also evaluated the difference in alpha diversity between all pairs of samples, termed here the delta alpha diversity, as a measure of community diversity variability both in collection method and within individuals over time. Samples collected with preservative had lower delta alpha diversity of Pielou evenness in individuals across days compared to unrelated individuals (Fig. 4b and Supplementary Fig. 2B). This further confirms our notion that the samples collected with preservative provide a more stable measurement of diversity over time.

### Differential ASV analyses

Although the sequenced microbiomes of samples collected with and without preservative were not identical, as illustrated by the variation in the diversity metrics, significant differences at the ASV level occurred in only 4% of the total number of ASVs, representing 27% percent by abundance differing between the collection methods (Supplementary Fig. 3A, B and Supplementary Table 1). When considering the absolute abundance, calculated using the *16S rRNA* gene qPCR copy numbers, there are similar patterns with 5% of the total number of ASVs (representing 25% by absolute abundance) differing between the collection methods (Supplementary Fig. 3C and Supplementary Table 2). Not surprisingly, the significantly different ASVs also were highly shared between the two analytic methods (Supplementary Fig. 3D).

Lastly, we performed analysis at the phylum level to understand potential large-scale taxonomic differences between the ASVs that differed between the two collection methods and those that did not. We found that the phylum composition was altered for the ASVs that differed between collection methods compared to those that did not (Fisher’s exact test, *P* < 0.001; Supplementary Table 3); however, 70% of the ASVs that were significantly different between collection methods were Gram negative bacteria, most of which were increased in abundance in the samples that were collected with preservative. This observation suggests that the preservative may be preserving bacteria that had been degrading in transit and/or in processing of the samples collected without preservative. In the absence of an a priori “gold standard”, the high concordance of ASVs detected provides evidence that preservative status had little impact on the detection of particular ASVs.

### Shotgun metagenomics

**Fig. 5:**
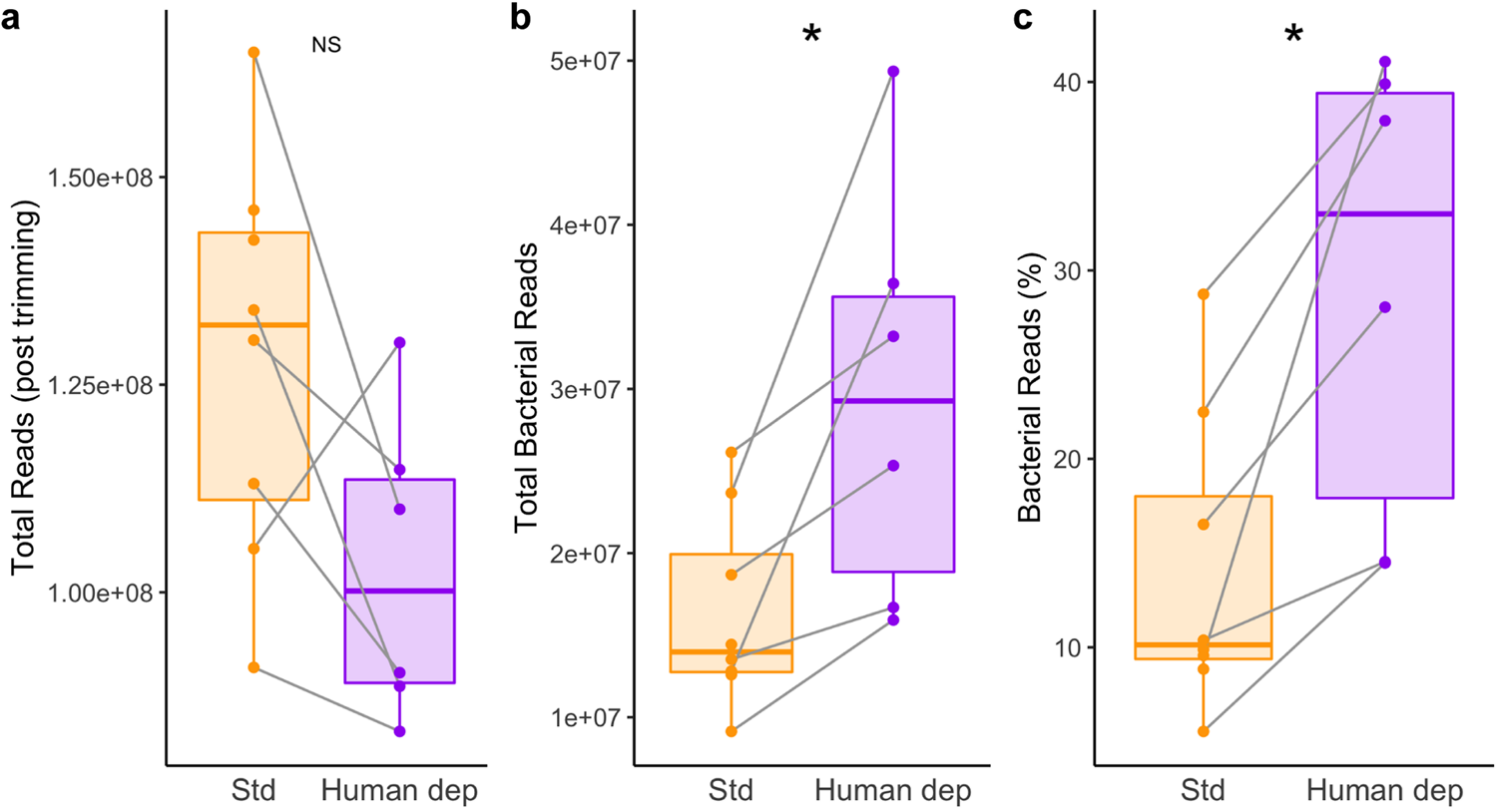
Bacterial DNA shotgun sequencing efficiency by extraction method (standard or with depletion of human DNA). **a** Total reads (post-trimming). **b** Total bacterial reads. **c** Bacterial reads as a percent of total reads; linear mixed effect model, *p < 0.05: NS = *p* > 0.05. Center line indicates median; box is IQR.

Next, we sought to assess whether depleting human DNA in the saliva samples would improve yield of bacterial DNA available for sequencing and whether it affected the representation of particular taxa and genes. Many techniques have been developed but none have been tested on samples containing DNA preservatives. To reduce the amount of human DNA present in shotgun sequencing, we examined human DNA depletion in a subset of five subjects, three of whom had samples on both day 1 and day 2, for a total of eight samples. Only those sample collected with preservative were sequenced (Fig. 1). Human DNA was depleted using the lysis propidium monoazide (lyPMA) technique^19^ prior to DNA extraction and compared to DNA extracted with standard procedures. Samples from one of the subjects (with samples on Day 1 and Day 2) had very low DNA concentrations in both DNA extractions and the human DNA depletion treatment DNA concentration was insufficient for sequencing. The standard extraction samples for this subject were sequenced and included in the analyses when paired tests between treatments were not performed. We observed that human DNA depletion yielded higher total and relative reads mapping to bacterial sequences compared to standard extraction methods (Fig. 5). The reduction in human DNA sequences in the samples is similar to previously reported results of samples frozen without cryogenic preservatives, which lowers human DNA concentrations compared to fresh saliva or cryogenically preserved saliva^19^.

**Fig. 6:**
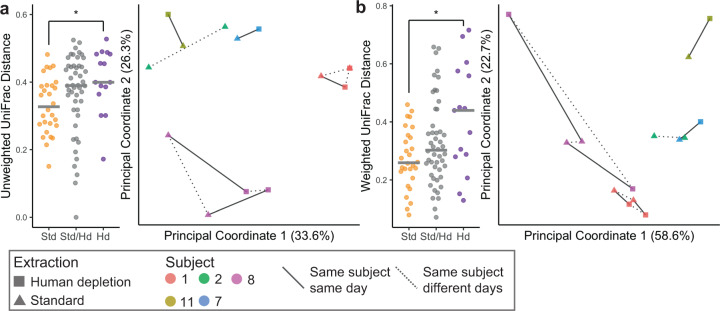
Phylogenetic beta diversity metrics of taxonomy determined from shotgun sequencing. Unweighted (**a**) and weighted (**b**) UniFrac of all samples according to the DNA extraction method. Left panels: Median distances in within-sample comparisons: standard vs standard (Std), human depletion vs human depletion (Hd) and across samples: standard vs human depletion (Std/Hd). Center line on plots indicates median. Right panels: PcoA plots of all samples by the extraction method; pairwise Wilcox test with FDR correction *q < 0.05. Lines connect specimens collected from the same subject on the same day (solid) or different days (dotted).

The beta diversities of the samples processed by the human depletion and standard methods did not significantly differ in any of the four metrics studied (Fig. 6 and Supplementary Fig. 4; PERMANOVA test *p* > 0.05). However, distances between human depletion samples was significantly higher than the distances between the standard method samples, indicating more measurable variability with the deeper bacterial sequencing in the former (Fig. 6 and Supplementary Fig. 4). However, in total, only 1 (0.3%) of 340 functional pathways and 6 (2.3%) of 265 species were significantly different in relative abundances between the two methods (Supplementary Fig. 5). These results provide further confirmation that overall pre-treatment to deplete human DNA before extraction does not significantly alter the bacterial composition of the sequenced metagenome.

**Fig. 7:**
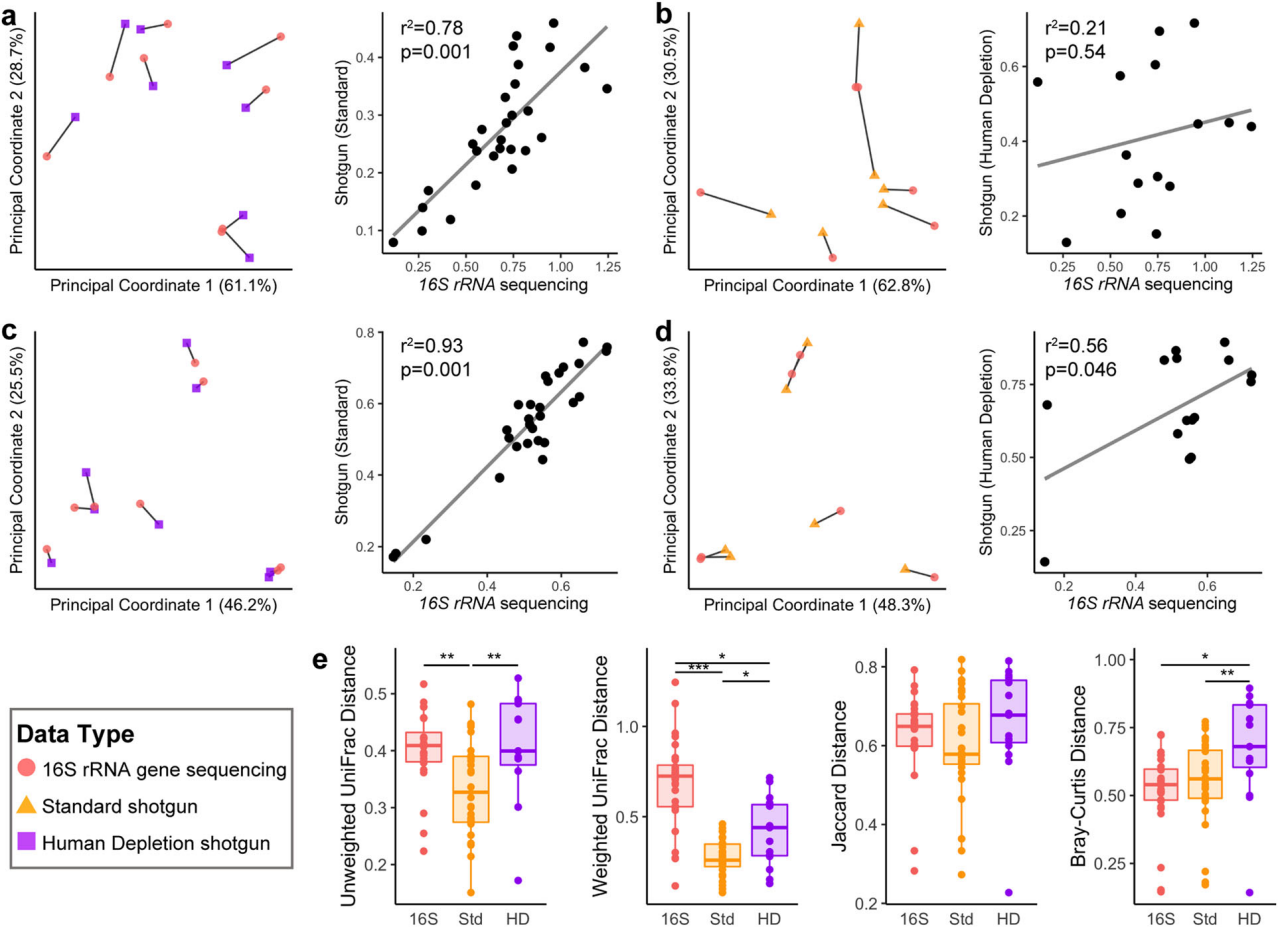
Procrustes analysis of *16S rRNA* gene and shotgun data of phylogenetic beta diversity. Weighted UniFrac (**a**) and Bray–Curtis (**c**) analysis comparing results from the *16S rRNA* gene sequencing and the standard extraction shotgun sequencing. Weighted UniFrac (**b**) and Bray–Curtis (**d**) analyses comparing results from the *16S rRNA* gene sequencing and human DNA depletion shotgun sequencing. Left panels: PCoA plots from procrustes analysis. Lines connect same samples across sequencing type. Right panels: Correlation plot from Mantel test comparing the two distance matrices. **e** Comparison of distances of all the shared samples between sequencing types. Center line on plots indicates median, box is IQR. *p < 0.05, **p < 0.01, ***p < 0.001.

Finally, we asked whether the community composition obtained as a result of *16S rRNA* gene sequencing deferred from that obtained from shotgun (metagenomic) with or without human DNA depletion. We found that all metrics comparing the *16S rRNA* gene sequencing results to the shotgun sequencing with or without human depletion were significantly correlated, with the exception of the weighted UniFrac analysis (Fig. 7a–d and Supplementary Fig, 6). Thus, the only appreciable variation was in relative abundances rather than overall composition. Interestingly, when comparing the distribution of distances within the eight samples between sequencing methods, the human DNA depletion shotgun samples had higher average beta diversity compared to the standard extraction shotgun samples, which was no different from the *16S rRNA* gene sequencing values in the unweighted metrics (Fig. 7e). This pattern also was more prominent in the phylogenetic measure of diversity, suggesting that the deeper sequencing in the human DNA depletion samples is preserving more phylogenetic diversity compared to the standard extraction.

In conclusion, we observed that kits used to collect saliva for the purpose of SARS-CoV-2 testing sufficiently preserve the microbiome DNA and are comparable to saliva collected without preservative. These results are consistent with and extend those of Lim et al.^17^, indicating that for saliva samples, collection method and extraction type minimally affect DNA amount and sequencing efficiency. The preservative did not hinder human DNA depletion, which was comparable to the previously observed success of samples frozen without preservative that had been used in the original development of the method^19^ which increased bacterial DNA reads in shotgun sequencing. Beyond methods, we report less variation within individuals over time compared to unrelated individuals, suggesting the longitudinal evaluation of subjects may provide perspectives into oral microbiome changes. These results make it practical to use saliva samples obtained for SARS-CoV-2 testing to examine the salivary microbiome in relation to SARS-CoV-2 infection, and the pathophysiologic changes it causes.

## Methods

### Study cohort

Subjects were recruited from a larger cohort following SARS-CoV-2 infection in hospital-affiliated workers^20^. The mean age of the participants was 47, and 72.7% of the participants were females. All patients were considered in good health and were negative for SARS-CoV-2 infection, based on concurrent qRT-PCR testing^20^. More information on study subjects is outlined in Table 1. The study protocol was approved by the Rutgers Institutional Review Board (protocol number Pro2020000679) and all subjects gave their written, informed consent.

### Sample collection

Each subject provided two saliva samples, one collected in an empty 15 mL conical tube, and the other collected using the Spectrum sDNA-1000 kit which includes a sample preservative. The order in which samples were collected was randomized between subjects. The six subjects who provided two longitudinal samples provided the second sample the day after their initial saliva sample, following the same collection protocol with samples collected in the same order as the previous day. At each collection, 2 mL of saliva were collected, and 1.5 mL of preservative added to the samples in the preservative-containing collection kit. To maintain a consistent dilution, 1.5 mL of sterile PBS was added to the samples collected without preservative. Samples were stored in −80 °C. Per biosafety guideline for working with samples that could potentially contain SARS-CoV-2, all collected saliva samples were heat decontaminated at 56°C for 60 min before handling.

### DNA extraction

DNA was extracted using the standard DNeasy PowerSoil Kit protocol (Qiagen) of saliva was used for extraction. Post-extraction DNA quantity and quality was measured using a Thermo NanoDrop 1000.

### *16S rRNA* gene quantification

*16S rRNA* gene was quantified with qPCR using SYBR green following kit instructions on a Roche LightCycler 480. The total reaction volume in each well was 20 µL containing 10 µL of SYBR green master mix, 2 µL of sample, 0.5 µL each of forward and reverse primers, and 7 µL of RNase-free water. The samples were run with pre-incubation at 95°C for 5 min and 45 amplification cycles of 95 °C for 10 s, 60 °C for 16 s, and 72 °C for 10 s, followed by a 1-min melting curve at 65 °C. Forward primer: 338 F-ACTCCTACGGGAGGCAGCAG, reverse primer: 518 R-ATTACCGCGGCTGCTGG

### *16S rRNA* sequencing library preparation and sequencing

The *16S rRNA* gene sequencing library was prepared using the Earth Microbiome Project (EMP) protocol^21^. In short, extracted saliva DNA was PCR-amplified with barcoded primers targeting the V4 region of *16S rRNA* gene according to the EMP 16S Illumina Amplicon protocol with the 515 F:806 R primer pairs. Control PBS and sample preservative that had undergone the same DNA extraction were also processed. Each PCR product was quantified using PicoGreen (Invitrogen), and an equal amounts (ng) of DNA from each sample were pooled and cleaned using the UltraClean PCR Clean-Up Kit (MoBio). All samples were prepared for sequencing in the same batch and run on the same sequencing run. DNA library preparations and sequencing reactions were conducted at GENEWIZ, Inc. (South Plainfield NJ, USA) using a MiSeq sequencing platform (Illumina, San Diego CA, USA). We performed 2 × 150 sequencing. After sequencing, we had a total of 11,875,289 read pairs with a mean of 29,688 read pairs per sample. Post-processing, we had a total of 11,032,587 single reads with a mean of 27,581 single reads per sample.

### *16S rRNA* gene sequencing bioinformatics and statistical analysis

Data were processed using QIIME2 version 2020.8 (ref. ^22^). We performed the analysis using a single end processing pipeline. In short, sequences were demultiplexed/denoised using the DADA2 q2 plugin^23^. Features were classified using the skLearn classifier in QIIME2 with a classifier that was pre-trained on Silva 138. The phylogenetic tree was built using the SEPP plugin and the Silva 128 reference tree^24^. Features that did not classify at the phylum level or were classified as mitochondria or chloroplast were filtered from the analysis. Samples were rarefied at 5164 reads. Diversity metrics and PERMANOVA statistical tests were calculated in QIIME2. All other statistics were calculated using the programming language R. To account for the repeat measures on subjects (across time and collection method), linear mixed effect models using the package nlme^25^ in R were used. When appropriate, we used Kruskal–Wallis rank sum test with Dunn’s post hoc test as necessary using base R. All visualizations were created using ggplot2 (ref. ^26^). Differential abundance tests were performed using MaAslin2 (ref. ^27^) with FDR correction. Features with a corrected *q* value < 0.05 were identified as significant.

### Shotgun metagenomic sequencing

Human DNA was depleted as described^19^. In short, 350 μL of saliva samples were spun down at 10,000 r.c.f. at 4 °C for 9 min. Supernatant was discarded and pellets were resuspended in 200 μL of sterile H_2_O and incubated at room temperature for 5 min, then 10 μL of a 0.2 mM PMA solution was added and samples were incubated in the dark for 5 min. Samples were then placed horizontally on ice <20 cm from a fluorescent light bulb (200 W, 2030 lumens, 3000 K) for 25 min, vertexing, and rotating every 5 min. Samples were stored at −20 °C until DNA extraction. DNA-depleted samples were then extracted using the Qiagen PowerSoil kit as described above. Duplicate samples from the standard extraction also were used for sequencing.

DNA library preparations and sequencing reactions were conducted at GENEWIZ, Inc. The NEB NextUltra DNA Library Preparation kit was used following the manufacturer’s recommendations (Illumina). Briefly, the genomic DNA was fragmented by acoustic shearing with a Covaris S220 instrument. The DNA was end-repaired and adenylated. Adapters were ligated after adenylation of the 3′ ends. Adapter-ligated DNA was indexed and enriched by limited cycle PCR. The DNA libraries were validated using TapeStation (Agilent Technologies, Palo Alto CA, USA), and quantified using a Qubit 2.0 Fluorometer. The DNA libraries were quantified by real-time PCR (Applied Biosystems, Carlsbad, CA, USA). The DNA libraries were loaded with equal amounts of DNA each on an Illumina HiSeq instrument according to the manufacturer’s instructions (Illumina). Sequencing was performed using a 2 × 150 paired end (PE) configuration; image analysis and base calling was conducted by the HiSeq Control Software (HCS) on the HiSeq instrument..bcl files were converted to fastq files and demultiplexed using bcl2fastq v. 2.17. After sequencing, we had a total of 1,808,032,554 single reads with a mean of 126,407,691 single reads per sample. Post-processing, we had a total of 277,043,256 microbial reads with a mean of 17,538,231 microbial reads per sample.

### Shotgun bioinformatics and statistical analysis

Sequences were trimmed and human reads were filtered out using the tool Kneaddata v0.10.0 (https://github.com/biobakery/kneaddata) using default parameters. Gene function and pathway analysis was conducted using HuMAnN3 v3.0.0a4 (ref. ^28^), with default parameters. Abundance of genes or pathways were renormalized to relative abundance using the HuMAnN3 utility script. Taxonomic identification was determined using MetaPhlAn3 v3.0.7 (ref. ^29^). Beta diversity metrics and analyses were calculated using QIIME2 version 2020.8 (ref. ^22^). Features were binned at the species taxonomic level prior to differential abundance analysis. Differential abundance tests were performed using MaAslin2 (ref. ^27^) with FDR correction. Features with a corrected *q* value <0.05 were identified as significant.

### Reporting summary

Further information on research design is available in the Nature Research Reporting Summary linked to this article.

## Supplementary information

Supplementary Information

Reporting Summary

## Data Availability

*16S rRNA* gene amplicon sequencing data are publicly available in EBI/ENA (https://www.ebi.ac.uk/ena) accession number ERP132364 and QIITA (https://qiita.ucsd.edu) Study ID 13807. Shotgun metagenomic sequencing data are available in EBI/ENA accession number PRJEB47800.

## Code availability

All analysis code is available upon request.
