# Supplementary material for: Assessing saliva microbiome collection and processing methods
Source: NPJ Biofilms Microbiomes. 2021 Nov 18;7:81. doi: 10.1038/s41522-021-00254-z (PMC8602330; doi:10.1038/s41522-021-00254-z)
Supplement: Supplementary file 1 — Supplementary Information [file 41522_2021_254_MOESM1_ESM.pdf]

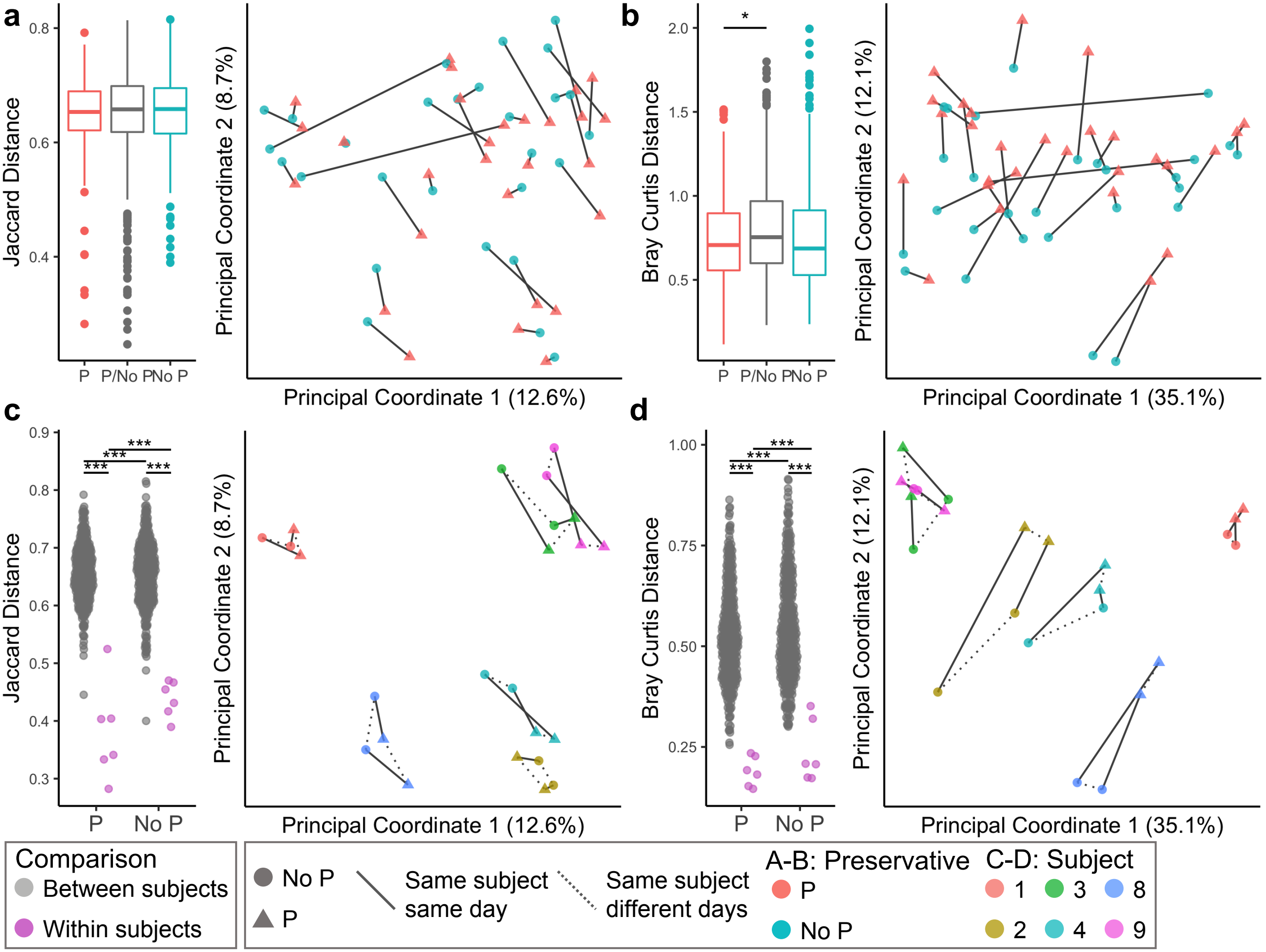

Supplementary Figure 1. Taxonomic beta diversity for 16S rRNA analyses of saliva samples by collection method and across time. Top panels: Jaccard (a) and Bray-Curtis (b) distance of all samples according to collection method. Left panels: Median (and IQR) distances in within-sample: Preservative samples only (P), non-preservative samples (No P), and across sample sample collection methods – preservative vs. non-preservative samples (P/No P). Right panels: PCoA plots of all samples by sample collection method. Bottom panels: Jaccard (c) and Bray-Curtis (d) UniFrac of the paired samples from 6 subjects sampled on two consecutive days and according to collection method. Left panels: Distance between unrelated subjects (gray) or within an individual across days (pink) separated by collection method: Preservative samples (P) and non-preservative samples (no P). Right panels: PCoA plot of all samples with only 6 multi-day subjects visualized. For all left panels, pairwise Wilcox test with FDR correction ** q < 0.01, *** q < 0.001. Lines connect specimens collected from the same subject on the same day (solid) or different days (dotted).

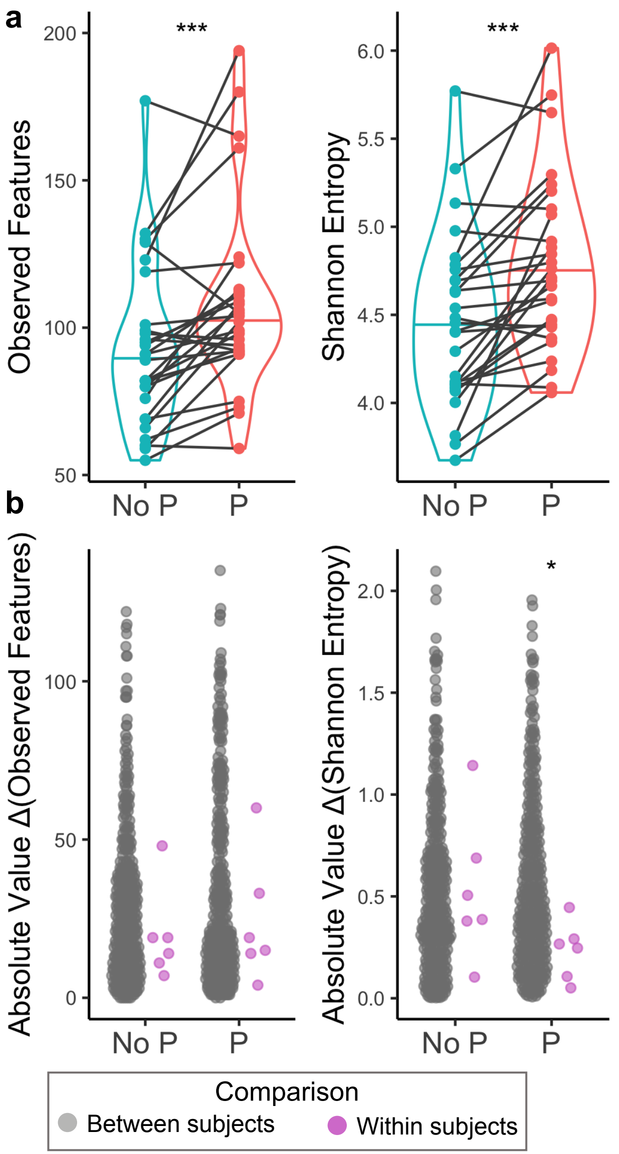

Supplementary Figure 2. Alpha diversity of samples, by collection method and over time. a. alpha diversity measures, based on Observed Features and Shannon Entropy, by sample collection with preservative (P) or not (No P). Lines connecting points indicate sample pairs. ** $p < 0.01$, *** $p < 0.001$; linear mixed effects model. b. Absolute value of the differences in alpha diversity between all unrelated subjects (pink circles), and longitudinal samples within the same subject (gray circles). * $p < 0.05$, ** $p < 0.01$; Kruskal-Wallis test.

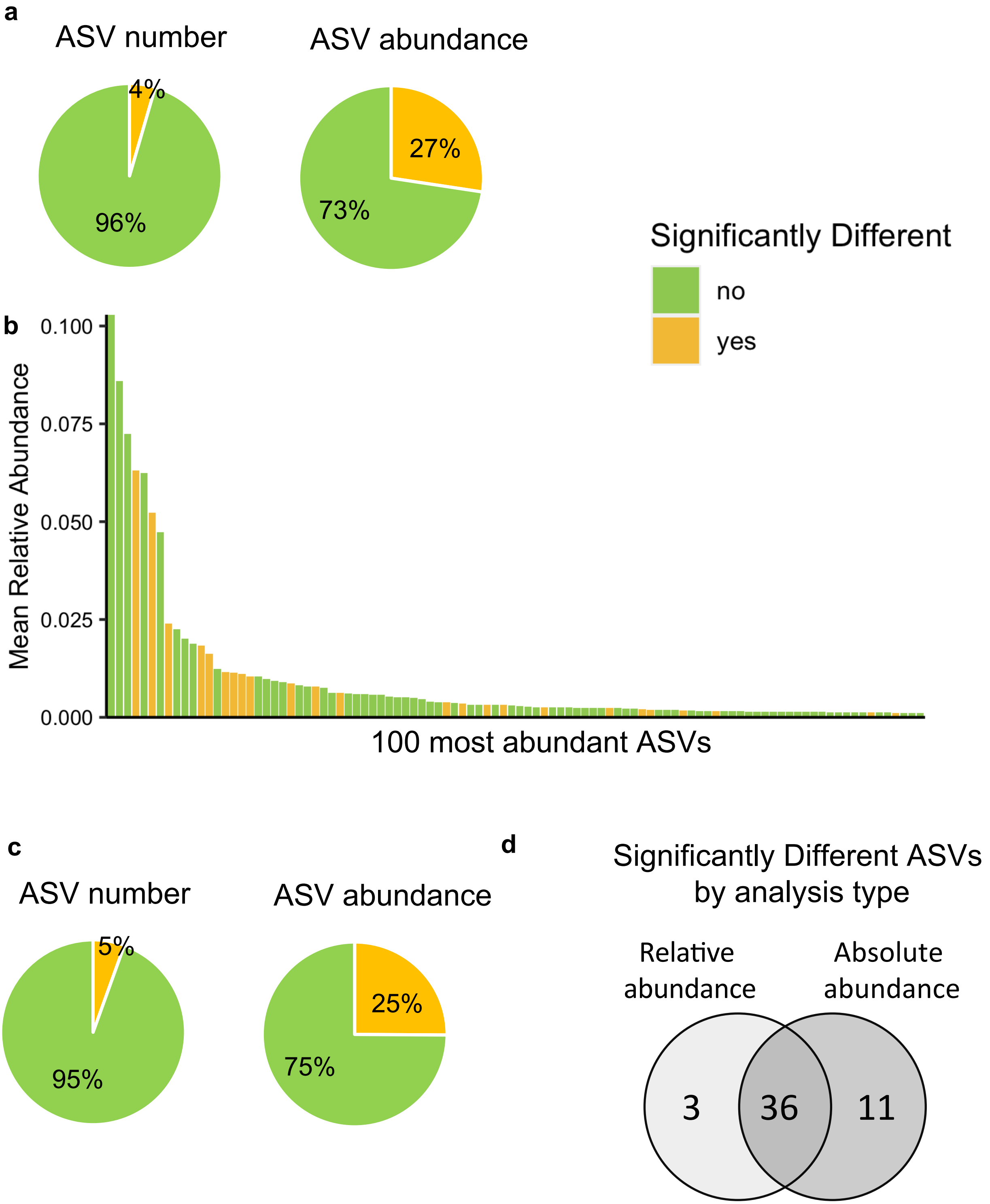

Supplementary Figure 3. Taxonomic differences between the sample collection methods with and without preservative based on 16S rRNA sequencing. a. Relative abundance analysis: proportion of the total 840 ASVs that show significantly different abundances between the two collection methods in the relative abundance analysis reported as the number of ASVs (left) and the share of the total abundance of ASVs (right). b. Ordering of the significantly different taxa within the 100 most abundant ASVs. c. Absolute abundance analysis: proportion of the total 840 ASVs that show significantly different abundances between the two collection methods when considering absolute abundance reported as the number of ASVs (left) and the share of the total abundance of ASVs (right). d. Venn diagram of ASVs that are significantly different between collection methods by analysis type - relative abundance analysis (a-b), absolute abundance analysis (c), or shared in both analyses

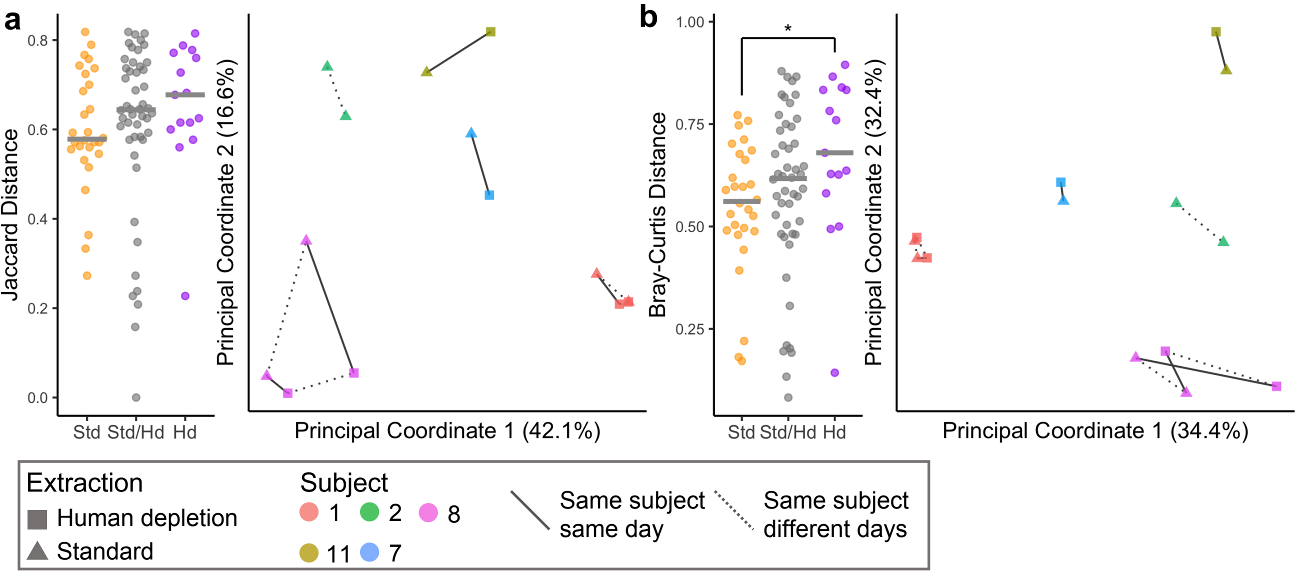

Supplementary Figure 4. Taxonomic beta diversity metrics of taxonomy determined from shotgun sequencing. Jaccard (a) and Bray-Curtis (b) distance of all samples according to DNA extraction method. Left panels: Median distances in within-sample: standard vs. standard (Std), human depletion vs human depletion (Hd) and across samples: standard vs human depletion (Std/Hd). Right panels: PCoA plots of all samples by extraction method; pairwise Wilcox test with FDR correction * $q < 0.05$. Lines connect specimens collected from the same subject on the same day (solid) or different days (dotted).

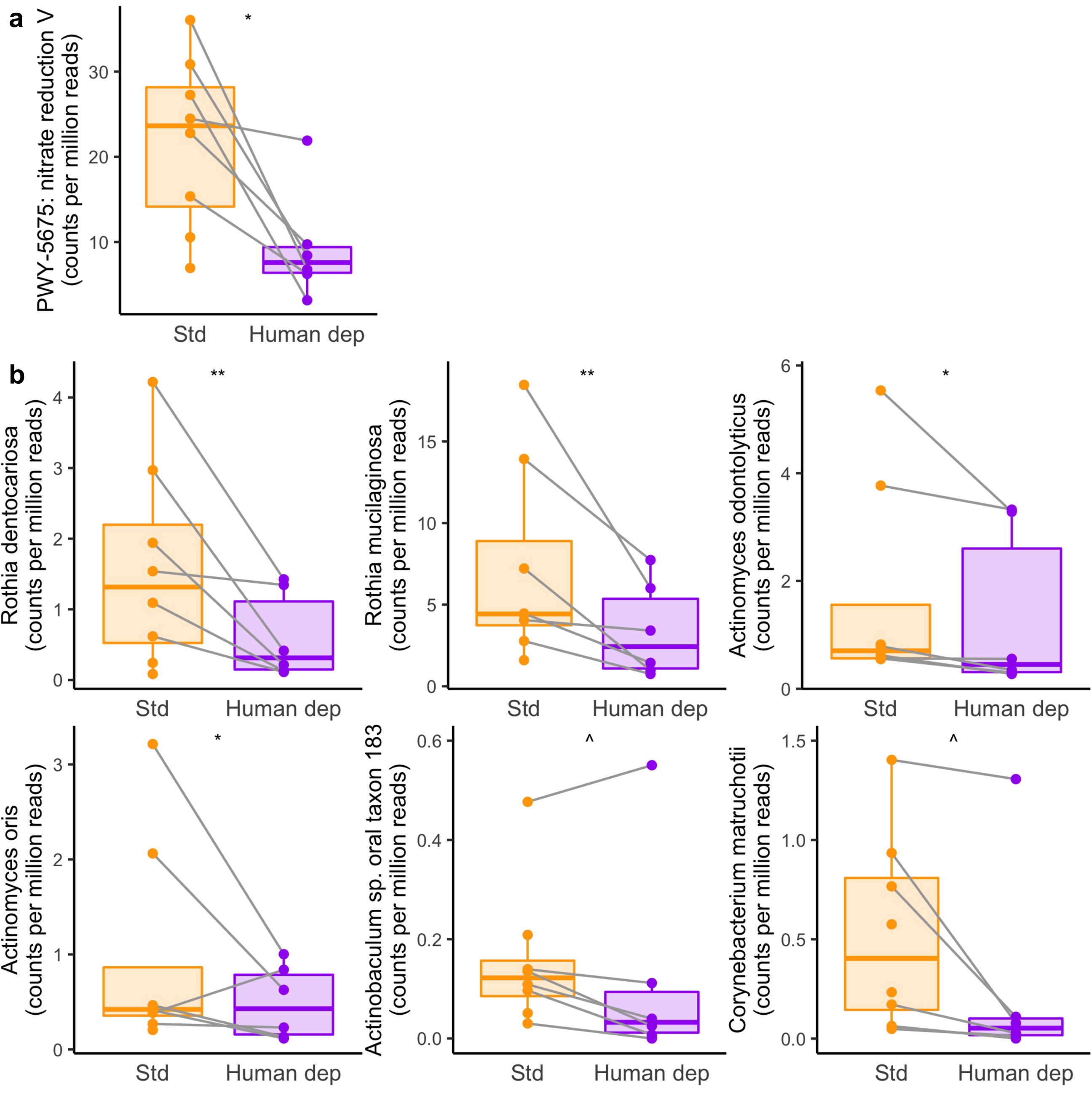

Supplementary Figure 5. Microbiome differences between extraction methods (Standard or with Depletion of human DNA). a. Only one (PWY-5675) of the total of 340 functional pathways that significantly differed between the two extraction methods. b. The six species (2.6%) among the total of 265 species that were significantly differing between extraction methods; linear mixed model with FDR correction, ^ $q < 0.1$, * $q < 0.05$, ** $q < 0.01$.

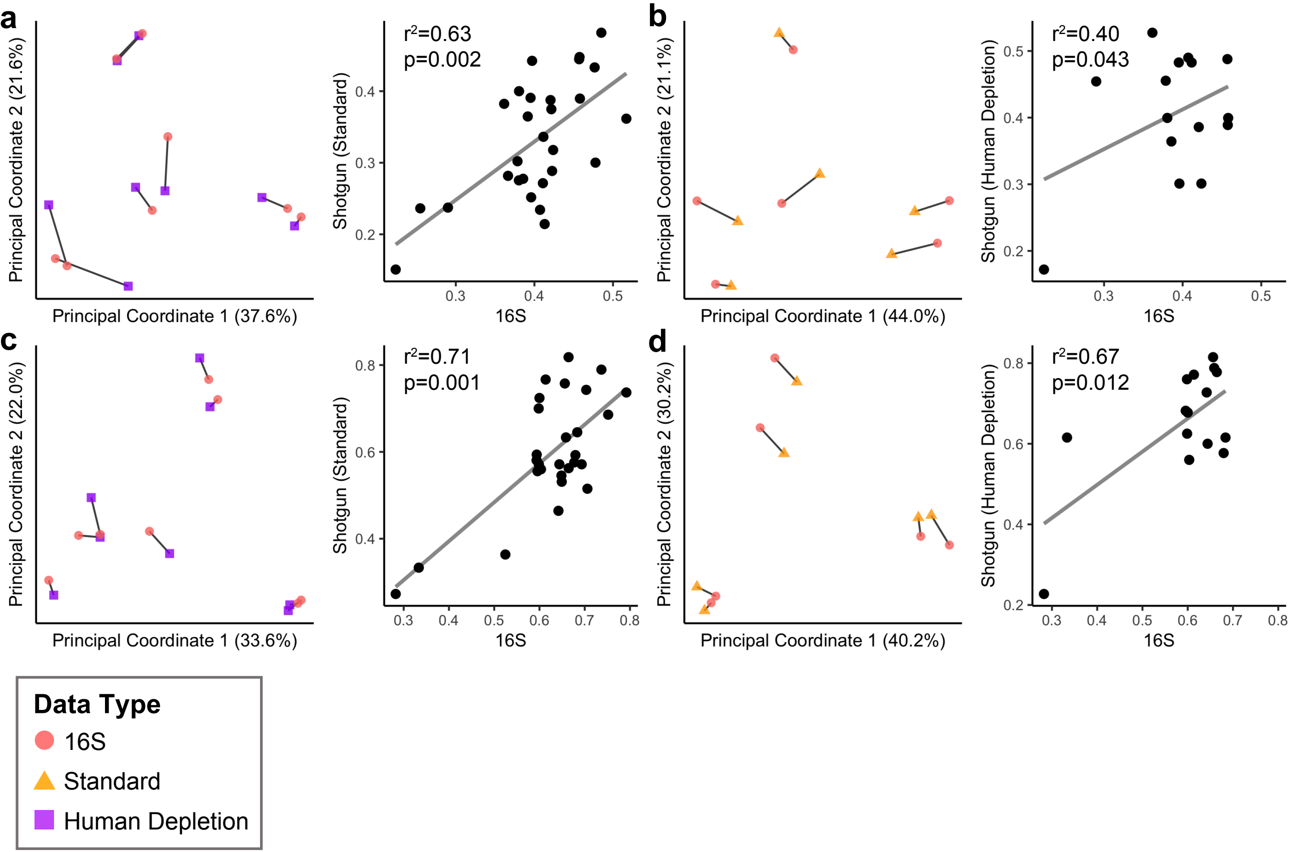

Supplementary Figure 6. Procrustes analysis of 16S and shotgun data of taxonomic beta diversity. Unweighted UniFrac (a) and Jaccard (c) analysis comparing results from the 16S sequencing and the standard extraction shotgun sequencing. Unweighted UniFrac (b) and Jaccard (d) analyses comparing results from the 16S sequencing and human depletion shotgun sequencing. Left panels: PCoA plots from procrustes analysis. Lines connect same samples across sequencing type. Right panels: Correlation plot from Mantel test comparing the two distance matrices.

Table S1. Comparison of taxa distinguished by alternative collection methods, based on Maaslin2 analysis of relative abundance, including features with >10% non-zero values. Features highlighted in yellow are significant

feature	coef*	stderr	pval	qval	annotation
X1a0bea5ccf9	0.60848003	0.06797516	2.54E-10	2.87E-08	d__Bacteria; p__Fusobacteriota; c__Fusobacteriia; o__Fusobacteriales; f__Fusobacteriaceae; g__Fusobacterium
c5adecb440a	0.63162641	0.06815145	1.30E-10	2.87E-08	d__Bacteria; p__Fusobacteriota; c__Fusobacteriia; o__Fusobacteriales; f__Fusobacteriaceae; g__Fusobacterium
X70dce344c1	0.58648364	0.08305753	4.29E-08	3.23E-06	d__Bacteria; p__Actinobacteriota; c__Actinobacteria; o__Corynebacteriales; f__Corynebacteriaceae; g__Corynebacterium; s__Corynebacterium_matruchotii
X446810522	0.44091854	0.07668637	1.87E-06	1.05E-04	s__Prevotella_nigrescens
ebfae3fd8d9d	0.54123041	0.10060174	5.96E-06	2.70E-04	d__Bacteria; p__Bacteroidota; c__Bacteroidia; o__Bacteroidales; f__Prevotellaceae; g__Prevotella
X0ae0be3085	0.44593183	0.08474563	9.22E-06	2.98E-04	d__Bacteria; p__Fusobacteriota; c__Fusobacteriia; o__Fusobacteriales; f__Fusobacteriaceae; g__Fusobacterium
ea0ff7a003ae	0.47243036	0.09045199	9.21E-06	2.98E-04	s__unidentified
X9ac5fec3962	-0.2417956	0.04646292	1.09E-05	3.08E-04	d__Bacteria; p__Actinobacteriota; c__Actinobacteria; o__Actinomycetales; f__Actinomycetaceae; g__Actinomyces; s__Schaalia_odontolytica
ce34506b9e1	0.20524559	0.04673599	1.08E-04	0.0027206	d__Bacteria; p__Campilobacterota; c__Campylobacteria; o__Campylobacterales; f__Campylobacteraceae; g__Campylobacter; s__unidentified
cce0f21cdf55	-0.2197612	0.05208831	1.89E-04	0.00388611	s__Rothia_mucilaginosa
X3cc657dfb4	0.36459938	0.08589717	1.78E-04	0.00388611	d__Bacteria; p__Bacteroidota; c__Bacteroidia; o__Flavobacteriales; f__Flavobacteriaceae; g__Capnocytophaga
X37b947daa0	0.34195861	0.08354714	2.45E-04	0.00396651	d__Bacteria; p__Fusobacteriota; c__Fusobacteriia; o__Fusobacteriales; f__Leptotrichiaceae; g__Leptotrichia; s__Leptotrichia_hongkongensis
X7b9a7ec76b	0.27517609	0.06600906	2.20E-04	0.00396651	d__Bacteria; p__Bacteroidota; c__Bacteroidia; o__Flavobacteriales; f__Weeksellaceae; g__Bergeyella
X82b92d7bd	-0.2448616	0.05936866	2.46E-04	0.00396651	d__Bacteria; p__Actinobacteriota; c__Actinobacteria; o__Micrococcales; f__Micrococcaceae; g__Rothia
fae8f7a4814d	0.29632622	0.07411189	3.50E-04	0.00527609	s__Prevotella_pallens
a159622ef88	0.41562336	0.1086762	5.54E-04	0.00782059	d__Bacteria; p__Firmicutes; c__Clostridia; o__Clostridia_UCG-014; f__Clostridia_UCG-014; g__Clostridia_UCG-014; s__Clostridiales_bacterium
ea6265476f0	0.40222906	0.10708082	7.13E-04	0.00947342	d__Bacteria; p__Fusobacteriota; c__Fusobacteriia; o__Fusobacteriales; f__Leptotrichiaceae; g__Leptotrichia; s__uncultured_bacterium
a8a8f3c4482	0.24352407	0.06697646	9.01E-04	0.01115615	d__Bacteria; p__Fusobacteriota; c__Fusobacteriia; o__Fusobacteriales; f__Fusobacteriaceae; g__Fusobacterium
X3ee072a59b	-0.1895703	0.0521772	9.38E-04	0.01115615	d__Bacteria; p__Actinobacteriota; c__Actinobacteria; o__Micrococcales; f__Micrococcaceae; g__Rothia
X8903ee9324	0.30789447	0.08601033	0.00120571	0.01328258	s__Prevotella_nanceiensis
X3bce60a047	0.26067102	0.07378836	0.00123422	0.01328258	d__Bacteria; p__Bacteroidota; c__Bacteroidia; o__Flavobacteriales; f__Flavobacteriaceae; g__Capnocytophaga
X9d4e168442	-0.1804435	0.05133326	0.00137068	0.01408061	d__Bacteria; p__Firmicutes; c__Bacilli; o__Staphylococcales; f__Gemellaceae; g__Gemella
X4c6bfea158	0.27394946	0.07888695	0.00157637	0.01484412	s__Prevotella_melaninogenica
fe02f7b94a8e	0.24462381	0.07154136	0.00157432	0.01484412	d__Bacteria; p__Fusobacteriota; c__Fusobacteriia; o__Fusobacteriales; f__Leptotrichiaceae; g__Leptotrichia; s__Leptotrichia_hofstadii
eb3043d3406	0.26747903	0.07887192	0.00174766	0.01579883	s__Prevotella_denticola
X7be9bac1b1	-0.122697	0.03651857	0.0020256	0.01760714	d__Bacteria; p__Firmicutes; c__Bacilli; o__Lactobacillales; f__Streptococcaceae; g__Streptococcus
b3d06445a91	-0.1103356	0.03353195	0.00244573	0.02047167	d__Bacteria; p__Firmicutes; c__Bacilli; o__Lactobacillales
X10953969f0	0.1687869	0.05248259	0.00273981	0.02135159	d__Bacteria; p__Firmicutes; c__Clostridia; o__Lachnospirales; f__Lachnospiraceae; g__Catonella
X0540bf08e9	-0.2534477	0.07806815	0.00266281	0.02135159	d__Bacteria; p__Actinobacteriota; c__Actinobacteria; o__Actinomycetales; f__Actinomycetaceae; g__Actinomyces; s__Actinomyces_graevenitzii
X219d2ac2e4	0.28309804	0.09175434	0.00400135	0.02832411	d__Bacteria; p__Firmicutes; c__Clostridia; o__Lachnospirales; f__Lachnospiraceae; g__Lachnoanaerobaculum
X13d195efab	0.1552119	0.05039758	0.00413582	0.02832411	d__Bacteria; p__Firmicutes; c__Bacilli; o__RF39; f__RF39; g__RF39; s__uncultured_bacterium
X5e947f6ca8	0.1830088	0.05950365	0.00413136	0.02832411	d__Bacteria; p__Fusobacteriota; c__Fusobacteriia; o__Fusobacteriales; f__Leptotrichiaceae; g__Leptotrichia
X8bbc849c70	0.13469786	0.04299014	0.00379334	0.02832411	d__Bacteria; p__Fusobacteriota; c__Fusobacteriia; o__Fusobacteriales; f__Fusobacteriaceae; g__Fusobacterium
fe87c08549ff	0.11765591	0.03879304	0.00480295	0.03192548	s__Prevotella_melaninogenica
aa5f922ae538	0.24881521	0.08406922	0.00567204	0.03662518	d__Bacteria; p__Fusobacteriota; c__Fusobacteriia; o__Fusobacteriales; f__Leptotrichiaceae; g__Leptotrichia; s__Leptotrichia_shahii
X39f89a63c1	0.22487197	0.07794622	0.00639044	0.04011774	d__Bacteria; p__Bacteroidota; c__Bacteroidia; o__Bacteroidales; f__Porphyromonadaceae; g__Porphyromonas; s__Porphyromonas_catoniae
X0eb193966	0.24553623	0.08536402	0.00716108	0.04374062	d__Bacteria; p__Patescibacteria; c__Gracilibacteria; o__Absconditabacteriales_(SR1); f__Absconditabacteriales_(SR1); g__Absconditabacteriales_(SR1); s__SR1_bacterium
X602510f333	0.17113795	0.05984242	0.00737079	0.04383683	s__Prevotella_melaninogenica
a6369fc8915	0.33331131	0.11967287	0.00871788	0.05051902	d__Bacteria; p__Bacteroidota; c__Bacteroidia; o__Bacteroidales; f__Porphyromonadaceae; g__Porphyromonas
df8284858d8	0.15181204	0.05501327	0.00935954	0.05159161	s__Prevotella_oris

X6e70d256d3	0.17311169	0.06271397	0.00933862	0.05159161	d__Bacteria; p__Bacteroidota; c__Bacteroidia; o__Bacteroidales; f__Prevotellaceae; g__Alloprevotella; s__uncultured_Bacteroidetes
e192c41a11d	0.17723572	0.06464319	0.0098918	0.05322733	s__Leptotrichia_sp.
X31b250015	0.24322991	0.08894055	0.01043214	0.05482936	d__Bacteria; p__Firmicutes; c__Clostridia; o__Peptococcales; f__Peptococcaceae; g__Peptococcus
X29e53aaab0	-0.2223044	0.08242611	0.0110122	0.05656268	d__Bacteria; p__Actinobacteriota; c__Actinobacteria; o__Micrococcales; f__Micrococcaceae; g__Rothia
X0c2876c22b	0.19834602	0.07418115	0.01132545	0.05687894	d__Bacteria; p__Fusobacteriota; c__Fusobacteriia; o__Fusobacteriales; f__Leptotrichiaceae; g__Leptotrichia; s__Leptotrichia_buccalis
cbe5861699e	0.13886261	0.05240292	0.01208111	0.05935501	s__Prevotella_pleuritidis
X0a69c0ff3a3	0.26764125	0.10153368	0.01257483	0.06046622	d__Bacteria; p__Firmicutes; c__Clostridia; o__Clostridia_UCG-014; f__Clostridia_UCG-014; g__Clostridia_UCG-014
ce33f26f3bd4	0.23513819	0.08981274	0.01326231	0.06132645	d__Bacteria; p__Fusobacteriota; c__Fusobacteriia; o__Fusobacteriales; f__Leptotrichiaceae; g__Leptotrichia
X8005a5fccd	0.14994111	0.05769527	0.01329644	0.06132645	d__Bacteria; p__Fusobacteriota; c__Fusobacteriia; o__Fusobacteriales; f__Leptotrichiaceae; g__Leptotrichia; s__Leptotrichia_goodfellowii
X6a2914e072	0.2126465	0.08207255	0.01416251	0.06401454	d__Bacteria; p__Bacteroidota; c__Bacteroidia; o__Bacteroidales; f__Prevotellaceae; g__Prevotella; s__Prevotella_sp.
f6852cab4e2	0.19503952	0.07642113	0.01561401	0.06786089	s__Prevotella_veroralis
d9b76ee51ff2	-0.136643	0.05339923	0.01548134	0.06786089	d__Bacteria; p__Firmicutes; c__Bacilli; o__Lactobacillales; f__Streptococcaceae; g__Streptococcus
X64bdd6a571	0.12164296	0.04782007	0.01612495	0.06812426	s__Prevotella_intermedia
c4007722892	0.17666492	0.06995091	0.01627748	0.06812426	s__Prevotella_pleuritidis
X6a66cd42df	0.21846975	0.08674294	0.01683187	0.06916367	s__Leptotrichia_sp.
ffa12a95bac2	0.26215277	0.10464499	0.01738919	0.07017781	s__Prevotella_pallens
X39d23124f2	0.14530059	0.05944223	0.01947525	0.07721767	d__Bacteria; p__Fusobacteriota; c__Fusobacteriia; o__Fusobacteriales; f__Leptotrichiaceae; g__Leptotrichia; s__Leptotrichia_wadei
X2fd80b8b34	0.18893042	0.07911279	0.02256946	0.08559466	s__Prevotella_genomosp.
X9af90fa49fa	0.2695519	0.11217035	0.02208025	0.08559466	s__Prevotella_nanceiensis
b29d6e91ae4	0.16573009	0.06932467	0.02272424	0.08559466	d__Bacteria; p__Proteobacteria; c__Gammaproteobacteria; o__Burkholderiales; f__Neisseriaceae
X49b6d0b43	0.1514552	0.06348638	0.02349303	0.08703975	s__Prevotella_intermedia
X72cbe4cdd9	0.29298808	0.12458003	0.02525003	0.09171863	s__Alloprevotella_rava
X252d0fd660	0.1718803	0.07354387	0.02557152	0.09171863	d__Bacteria; p__Actinobacteriota; c__Actinobacteria; o__Bifidobacteriales; f__Bifidobacteriaceae; g__Scardovia; s__Scardovia_wiggsiae
X5ba90da636	0.18969551	0.08161315	0.02597342	0.09171863	d__Bacteria; p__Fusobacteriota; c__Fusobacteriia; o__Fusobacteriales; f__Leptotrichiaceae; g__Leptotrichia
X491769136	0.1589063	0.06908726	0.02768009	0.09624154	s__Leptotrichia_sp.
f3cac486ad34	0.13769177	0.06157495	0.03209028	0.10665299	s__Prevotella_buccae
X76075eede2	0.13596401	0.06049093	0.03169073	0.10665299	g__Aggregatibacter
af684f96f945	0.18922031	0.08456292	0.03182351	0.10665299	s__Alloprevotella_tannerae
X1fdb57d940	0.14108634	0.06364959	0.03377789	0.11063482	d__Bacteria; p__Bacteroidota; c__Bacteroidia; o__Bacteroidales; f__Porphyromonadaceae; g__Porphyromonas; s__uncultured_bacterium
X7d4bc1c15b	-0.1284395	0.05851587	0.03555656	0.11479691	d__Bacteria; p__Firmicutes; c__Bacilli; o__Lactobacillales; f__Streptococcaceae; g__Streptococcus
e796676dcb0	0.08596608	0.03952674	0.03686349	0.11734013	d__Bacteria; p__Actinobacteriota; c__Coriobacteriia; o__Coriobacteriales; f__Atopobiaceae; g__Atopobium
d4c49968a5f	0.05658784	0.02624482	0.03849605	0.12083482	d__Bacteria; p__Firmicutes; c__Clostridia; o__Peptostreptococcales-Tissierellales; f__Peptostreptococcaceae; g__Peptoanaerobacter; s__Peptoanaerobacter_stomatis
b7a041d2927	0.14909763	0.0702912	0.04142602	0.12774566	g__Campylobacter
cca22a273fa1	-0.1338748	0.06313902	0.04182822	0.12774566	d__Bacteria; p__Firmicutes; c__Negativicutes; o__Veillonellales-Selenomonadales; f__Veillonellaceae; g__Megasphaera; s__Megasphaera_micronuciformis
X502ecbbaeb	0.0953819	0.04523713	0.04385919	0.13216235	s__Prevotella_oralis
X10a9a08874	-0.1348851	0.0648446	0.04535277	0.13486482	d__Bacteria; p__Proteobacteria; c__Gammaproteobacteria; o__Pasteurellales; f__Pasteurellaceae; g__Actinobacillus
d4dc4e8d0ff0	0.22686151	0.10955472	0.04611816	0.13535979	s__uncultured_bacterium
X499137328	0.11668617	0.05663063	0.04737467	0.13726506	d__Bacteria; p__Firmicutes; c__Clostridia; o__Lachnospirales; f__Lachnospiraceae; g__Oribacterium
f757847365a	0.17491411	0.08667251	0.05131375	0.14679629	d__Bacteria; p__Bacteroidota; c__Bacteroidia; o__Bacteroidales; f__Tannerellaceae; g__Tannerella
X6fd5fcf5367	0.22495381	0.11187867	0.05255269	0.14761477	s__uncultured_bacterium
aadada17f552	0.12419612	0.0617331	0.05290618	0.14761477	d__Bacteria; p__Firmicutes; c__Clostridia; o__Lachnospirales; f__Lachnospiraceae; g__Butyrivibrio
b8c6906ea99	-0.1264683	0.06415224	0.05739696	0.15819162	d__Bacteria; p__Actinobacteriota; c__Actinobacteria; o__Micrococcales; f__Micrococcaceae; g__Rothia
f434ebfe13e3	0.1550403	0.07920316	0.05889282	0.16035876	d__Bacteria; p__Firmicutes; c__Negativicutes; o__Veillonellales-Selenomonadales; f__Veillonellaceae; g__Veillonella; s__Veillonella_sp.
X6c83fec6e8	-0.1100419	0.05674802	0.06135738	0.16508058	d__Bacteria; p__Firmicutes; c__Negativicutes; o__Veillonellales-Selenomonadales; f__Veillonellaceae; g__Veillonella

X47c98abffb7	-0.1752045	0.09221765	0.06724692	0.1746874	s__Streptococcus_anginosus
X9e53a677b1	0.19990573	0.10526581	0.06677419	0.1746874	d__Bacteria; p__Patescibacteria; c__Gracilibacteria; o__Absconditabacteriales_(SR1); f__Absconditabacteriales_(SR1); g__Absconditabacteriales_(SR1); s__SR1_bacterium
e2c3ff4f6471	0.05010351	0.02637012	0.06650939	0.1746874	g__Bifidobacterium
X601dcc5af9	0.12768881	0.06812122	0.07005704	0.17789765	d__Bacteria; p__Firmicutes; c__Negativicutes; o__Veillonellales-Selenomonadales; f__Selenomonadaceae
X68db566a60	-0.1443092	0.07705239	0.07004039	0.17789765	d__Bacteria; p__Actinobacteriota; c__Actinobacteria; o__Actinomycetales; f__Actinomycetaceae; g__Actinomyces
X1986030e3	0.13006186	0.0713155	0.07694314	0.19321276	d__Bacteria; p__Proteobacteria; c__Gammaproteobacteria; o__Pasteurellales; f__Pasteurellaceae; g__Haemophilus
X3a22f9a8f97	0.13887973	0.07645358	0.07871492	0.1933649	s__Alloprevotella_tannerae
X6c1f3bf3e21	0.16369944	0.08997778	0.07800596	0.1933649	d__Bacteria; p__Firmicutes; c__Clostridia; o__Peptostreptococcales-Tissierellales; f__Peptostreptococcales-Tissierellales; g__Parvimonas
X1f4c8ba04b	-0.0873214	0.04828445	0.08002296	0.19446439	d__Bacteria; p__Actinobacteriota; c__Actinobacteria; o__Actinomycetales; f__Actinomycetaceae; g__Actinomyces; s__Schaalia_odontolytica
X6362083e0	0.18360815	0.10274352	0.08349791	0.2007503	d__Bacteria; p__Proteobacteria; c__Gammaproteobacteria; o__Cardiobacteriales; f__Cardiobacteriaceae; g__Cardiobacterium; s__uncultured_bacterium
da19337673	0.08217175	0.04746004	0.09266968	0.22045628	s__uncultured_bacterium
X44598e75a1	0.12067309	0.07075099	0.09743334	0.22937433	s__unidentified
X38e3b7abf4	0.09929983	0.0588325	0.10166678	0.23364696	d__Bacteria; p__Fusobacteriota; c__Fusobacteriia; o__Fusobacteriales; f__Fusobacteriaceae; g__Fusobacterium
ffb88213324	0.08069682	0.04777168	0.1008251	0.23364696	d__Bacteria; p__Firmicutes; c__Bacilli; o__RF39; f__RF39; g__RF39; s__Firmicutes_oral
X7217df85b1	0.21907395	0.13040017	0.10234977	0.23364696	s__unidentified
d44eca03358	0.02793722	0.01670266	0.10367659	0.23430909	d__Bacteria; p__Proteobacteria; c__Gammaproteobacteria; o__Burkholderiales; f__Neisseriaceae
X641e0a0923	0.06660775	0.0407134	0.11105729	0.24850443	s__Streptococcus_anginosus
X984ef97167	0.09532522	0.05895475	0.11567323	0.25380727	s__Oribacterium_parvum
X685b8609f6	0.14177798	0.08762017	0.11482022	0.25380727	s__Prevotella_shahii
X5a0df24ab3	-0.0924596	0.05782864	0.11939379	0.25781252	d__Bacteria; p__Actinobacteriota; c__Coriobacteriia; o__Coriobacteriales; f__Atopobiaceae; g__Atopobium; s__uncultured_bacterium
e0f64c7eb3c	-0.0990563	0.06219141	0.11978015	0.25781252	d__Bacteria; p__Firmicutes; c__Clostridia; o__Peptostreptococcales-Tissierellales; f__Anaerovoracaceae; g__[Eubacterium]_nodatum_group; s__[Eubacterium]_nodatum
ae92f7ff4fd72	0.16517725	0.10451918	0.12384115	0.26403868	s__Eubacterium_sp.
X4904c5ac70	0.09462734	0.06133192	0.13088463	0.2760446	d__Bacteria; p__Bacteroidota; c__Bacteroidia; o__Flavobacteriales; f__Flavobacteriaceae; g__Capnocytophaga
X95348b8c1	0.09029893	0.0584064	0.13191512	0.2760446	d__Bacteria; p__Firmicutes; c__Negativicutes; o__Veillonellales-Selenomonadales; f__Veillonellaceae; g__Veillonella
X33206a230	-0.08604	0.05676441	0.13986477	0.28999484	d__Bacteria; p__Proteobacteria; c__Gammaproteobacteria; o__Pasteurellales; f__Pasteurellaceae; g__Actinobacillus; s__Haemophilus_parahaemolyticus
X26800fe52e	0.24736358	0.16497293	0.14315577	0.29412004	d__Bacteria; p__Bacteroidota; c__Bacteroidia; o__Bacteroidales; f__Prevotellaceae; g__Alloprevotella; s__uncultured_Bacteroidetes
X6d86c9301	0.06273941	0.04262837	0.15024487	0.30317268	s__Prevotella_aurantiaca
X19daa442e7	0.02700277	0.01829692	0.14948589	0.30317268	d__Bacteria; p__Firmicutes; c__Negativicutes; o__Veillonellales-Selenomonadales; f__Veillonellaceae
X510f6fbe9d	0.09129493	0.06245493	0.15311467	0.30622934	d__Bacteria; p__Firmicutes; c__Clostridia; o__Peptostreptococcales-Tissierellales; f__Anaerovoracaceae; g__[Eubacterium]_brachy_group; s__Eubacterium_brachy
f83bc32e60e	-0.0313386	0.02153355	0.15522181	0.30772042	g__Bifidobacterium
X1c138843b	0.09675761	0.06760547	0.16223051	0.31606978	d__Bacteria; p__Bacteroidota; c__Bacteroidia; o__Flavobacteriales; f__Flavobacteriaceae; g__Capnocytophaga; s__uncultured_Capnocytophaga
X15dd83460	0.10783364	0.07516308	0.16106604	0.31606978	g__Aggregatibacter
X3827b3842	-0.1171455	0.08303277	0.16783615	0.32419632	d__Bacteria; p__Actinobacteriota; c__Actinobacteria; o__Actinomycetales; f__Actinomycetaceae; g__Actinomyces
cf4e43ffaa77	0.11003886	0.08132003	0.18526925	0.35483771	d__Bacteria; p__Firmicutes; c__Clostridia; o__Peptostreptococcales-Tissierellales; f__Peptostreptococcaceae; g__Filifactor; s__Filifactor_alocis
X2c1a555f04	0.0538259	0.04102652	0.19863634	0.37724212	g__Campylobacter
X1bb583679	0.12283263	0.09460703	0.20296656	0.38225369	d__Bacteria; p__Firmicutes; c__Negativicutes; o__Veillonellales-Selenomonadales; f__Selenomonadaceae; g__Selenomonas; s__Selenomonas_sputigena
X4458477e8	0.11288734	0.08709714	0.20471934	0.38236835	s__Prevotella_oris
d79a701e5a4	0.07961016	0.06321951	0.21714411	0.40225056	s__Prevotella_saccharolytica
b4f5c5ff1599	0.06195754	0.04986001	0.22320559	0.40355571	g__Selenomonas
X8e058482fc	0.0657788	0.05285553	0.222119	0.40355571	s__Eubacterium_sp.
fdcd6808ef82	0.11402782	0.09156546	0.22162367	0.40355571	d__Bacteria; p__Proteobacteria; c__Gammaproteobacteria; o__Burkholderiales; f__Neisseriaceae; g__Neisseria

f9d1dd7ca23	0.07730548	0.06664448	0.25443209	0.45276892	s__Prevotella_shahii
X78e469b5fd	-0.0369133	0.03172812	0.25330647	0.45276892	d__Bacteria; p__Firmicutes; c__Clostridia; o__Lachnospirales; f__Lachnospiraceae; g__Oribacterium
f55030f7203	0.07331335	0.06367834	0.25666846	0.45318026	d__Bacteria; p__Bacteroidota; c__Bacteroidia; o__Bacteroidales; f__Prevotellaceae; g__Alloprevotella; s__uncultured_eubacterium
aacaf5f360c1	-0.1041902	0.09188046	0.26492638	0.46413459	d__Bacteria; p__Firmicutes; c__Negativicutes; o__Veillonellales-Selenomonadales; f__Selenomonadaceae; g__Selenomonas; s__Selenomonas_artemidis
ce4de2da787	0.08851123	0.08086205	0.28043508	0.4875256	s__Prevotella_oulorum
X3f6c45001f	-0.0569939	0.05250401	0.28598216	0.4933738	d__Bacteria; p__Proteobacteria; c__Gammaproteobacteria; o__Burkholderiales; f__Neisseriaceae; g__Neisseria
b6a0f2aee9b	0.0590509	0.05479753	0.28841176	0.49379589	d__Bacteria; p__Fusobacteriota; c__Fusobacteriia; o__Fusobacteriales; f__Leptotrichiaceae; g__Leptotrichia
X49757d4da8	0.03417751	0.03196218	0.29352103	0.49504294	d__Bacteria; p__Proteobacteria; c__Gammaproteobacteria; o__Burkholderiales; f__Neisseriaceae; g__Alysiella
d8f2651dc1a	0.05482005	0.05131285	0.29290991	0.49504294	d__Bacteria; p__Bacteroidota; c__Bacteroidia; o__Flavobacteriales; f__Flavobacteriaceae; g__Capnocytophaga
X447ee7f88c	0.04252497	0.04099849	0.30686849	0.50255274	d__Bacteria; p__Fusobacteriota; c__Fusobacteriia; o__Fusobacteriales; f__Fusobacteriaceae; g__Fusobacterium
X0755e2d58	0.09405359	0.09050606	0.30617327	0.50255274	d__Bacteria; p__Proteobacteria; c__Gammaproteobacteria; o__Burkholderiales; f__Neisseriaceae; g__Kingella; s__uncultured_bacterium
X41a360fde2	0.05390697	0.05172552	0.30384967	0.50255274	d__Bacteria; p__Firmicutes; c__Bacilli; o__Mycoplasmatales; f__Mycoplasmataceae; g__Mycoplasma; s__Mycoplasmopsis_arginini
X2731574ed2	0.04173511	0.03985818	0.30216591	0.50255274	s__Prevotella_maculosa
X29bb5fa7f4	0.08684314	0.08462249	0.31233362	0.50419569	d__Bacteria; p__Firmicutes; c__Negativicutes; o__Veillonellales-Selenomonadales; f__Veillonellaceae; g__Veillonella
X0d672b487	0.08513363	0.08271082	0.31179861	0.50419569	d__Bacteria; p__Bacteroidota; c__Bacteroidia; o__Bacteroidales; f__Prevotellaceae; g__Prevotella; s__Prevotella_sp.
X9d863926e	0.07851863	0.07827068	0.32353381	0.50426649	d__Bacteria; p__Proteobacteria; c__Gammaproteobacteria; o__Burkholderiales; f__Comamonadaceae; g__Comamonas; s__Ottowia_sp.
X1d8aede440	0.03578957	0.03544742	0.3211817	0.50426649	s__Firmicutes_oral
def8acc0fc88	-0.0886428	0.08776767	0.31995935	0.50426649	d__Bacteria; p__Firmicutes; c__Bacilli; o__Erysipelotrichales; f__Erysipelotrichaceae; g__Solobacterium; s__Solobacterium_moorei
X643cef76cf1	0.0455821	0.04513752	0.32074871	0.50426649	d__Bacteria; p__Proteobacteria; c__Gammaproteobacteria; o__Burkholderiales; f__Neisseriaceae
X5310d435fd	0.03837347	0.03816224	0.32239296	0.50426649	s__Prevotella_oulorum
ae0c6cee8a6	-0.1186578	0.11948201	0.32801053	0.50774233	d__Bacteria; p__Firmicutes; c__Clostridia; o__Peptostreptococcales-Tissierellales; f__Anaerovoracaceae; g__[Eubacterium]_nodatum_group
X5b1b3a87b	0.05584189	0.05737283	0.33689241	0.51794343	d__Bacteria; p__Spirochaetota; c__Spirochaetia; o__Spirochaetales; f__Spirochaetaceae; g__Treponema; s__Treponema_socranskii
X9e8e56f06c	-0.0715016	0.07632789	0.35565657	0.54309719	d__Bacteria; p__Actinobacteriota; c__Actinobacteria; o__Actinomycetales; f__Actinomycetaceae; g__Actinomyces
eb44896f88d	-0.0946832	0.10498166	0.3737427	0.55937649	d__Bacteria; p__Firmicutes; c__Clostridia; o__Lachnospirales; f__Lachnospiraceae; g__Stomatobaculum; s__uncultured_bacterium
X95f77f9e6df	0.04590248	0.05081402	0.37241593	0.55937649	d__Bacteria; p__Firmicutes; c__Negativicutes; o__Veillonellales-Selenomonadales; f__Veillonellaceae
f64be8a0bee	-0.0120765	0.01335732	0.37275354	0.55937649	d__Bacteria; p__Firmicutes; c__Negativicutes; o__Veillonellales-Selenomonadales; f__Veillonellaceae; g__Veillonella
X3e3cbcd987	0.02423769	0.02790274	0.39140794	0.5819618	s__Prevotella_aurantiaca
d2e36e4099b	0.04456457	0.05223726	0.40099452	0.58847248	d__Bacteria; p__Patescibacteria; c__Saccharimonadia; o__Saccharimonadales; f__Saccharimonadaceae; g__Candidatus_Saccharimonas; s__uncultured_bacterium
ea322a31ace	0.05157844	0.06059923	0.40051721	0.58847248	d__Bacteria; p__Firmicutes; c__Clostridia; o__Peptostreptococcales-Tissierellales; f__Peptostreptococcaceae; g__[Eubacterium]_yurii_group; s__uncultured_bacterium
X25def15fb42	-0.0764126	0.09088692	0.406328	0.58865467	g__Akkermansia
X151691808	0.07745549	0.09189992	0.4045949	0.58865467	g__Campylobacter
X28a3212dff	0.07979988	0.09631746	0.41347774	0.59519726	d__Bacteria; p__Patescibacteria; c__Gracilibacteria; o__Absconditabacteriales_(SR1); f__Absconditabacteriales_(SR1); g__Absconditabacteriales_(SR1); s__SR1_bacterium
be4ccff9dd8d	0.06137335	0.07469582	0.41734621	0.59696356	d__Bacteria; p__Bacteroidota; c__Bacteroidia; o__Flavobacteriales; f__Flavobacteriaceae; g__Capnocytophaga
e1dc096664	-0.0539216	0.06901745	0.4396651	0.62493278	d__Bacteria; p__Actinobacteriota; c__Actinobacteria; o__Actinomycetales; f__Actinomycetaceae; g__Actinomyces; s__Actinomyces_dentalis
d44d4b8a0a3	0.0428082	0.05626826	0.45259895	0.63737606	d__Bacteria; p__Proteobacteria; c__Gammaproteobacteria; o__Burkholderiales; f__Neisseriaceae; g__Neisseria; s__Neisseria_oralis
fe30c2d2ad2	0.034711	0.04580601	0.45405994	0.63737606	d__Bacteria; p__Bacteroidota; c__Bacteroidia
X82d2a5f97a	0.06411918	0.08525476	0.45780862	0.63867128	s__Prevotella_salivae
X2d750a5907	0.03732753	0.05023279	0.46222356	0.6397422	s__Prevotella_oris
c1b636001e0	-0.0459006	0.06199191	0.46423771	0.6397422	d__Bacteria; p__Firmicutes; c__Negativicutes; o__Veillonellales-Selenomonadales; f__Veillonellaceae; g__Veillonella

X29838c8386	0.05631147	0.07676493	0.46809796	0.64115235	d__Bacteria; p__Actinobacteriota; c__Actinobacteria; o__Actinomycetales; f__Actinomycetaceae; g__F0332; s__uncultured_bacterium
f1dfcb23cf4d	0.02724387	0.03778638	0.47586999	0.64563646	d__Bacteria; p__Synergistota; c__Synergistia; o__Synergistales; f__Synergistaceae; g__Fretibacterium
X93233cf6a3	-0.0739555	0.10317028	0.4779472	0.64563646	d__Bacteria; p__Firmicutes; c__Bacilli; o__Lactobacillales; f__Streptococcaceae; g__Streptococcus
X2f297c39eca	0.0811082	0.11353141	0.47994214	0.64563646	d__Bacteria; p__Firmicutes; c__Clostridia; o__Peptostreptococcales-Tissierellales; f__Peptostreptococcales-Tissierellales; g__Parvimonas
cdce04d738e	0.06789358	0.09617866	0.48532938	0.64902036	g__Aggregatibacter
X5e6aa5e331	-0.040841	0.06016803	0.50125101	0.66636899	d__Bacteria; p__Firmicutes; c__Clostridia; o__Lachnospirales; f__Lachnospiraceae
X46aaf5ecb5c	0.07777665	0.11903503	0.51796313	0.68123362	d__Bacteria; p__Firmicutes; c__Clostridia; o__Lachnospirales; f__Lachnospiraceae; g__Oribacterium
X9ead73ac54	0.0920586	0.14094503	0.51846099	0.68123362	d__Bacteria; p__Proteobacteria; c__Gammaproteobacteria; o__Burkholderiales; f__Burkholderiaceae; g__Lautropia; s__Lautropia_sp.
X8bf1d2fa2b5	-0.0439007	0.07084657	0.53920394	0.70439359	g__Centipeda
de8a1c0d12e	0.0207264	0.0340974	0.54729072	0.71084886	d__Bacteria; p__Bacteroidota; c__Bacteroidia; o__Flavobacteriales; f__Flavobacteriaceae; g__Capnocytophaga; s__uncultured_Capnocytophaga
f7f2cea1e0c0	0.03669902	0.06455885	0.57369397	0.73667521	d__Bacteria; p__Firmicutes; c__Bacilli; o__Lactobacillales; f__Streptococcaceae; g__Streptococcus
ebd6c1d23c5	0.02395982	0.04185563	0.57096762	0.73667521	d__Bacteria; p__Proteobacteria; c__Gammaproteobacteria; o__Burkholderiales; f__Neisseriaceae; g__Neisseria
X83aed15ae1	-0.0350759	0.0624355	0.57818013	0.7382413	d__Bacteria; p__Actinobacteriota; c__Actinobacteria; o__Micrococcales; f__Micrococcaceae; g__Rothia
X5d6f77f2bcb	0.026447	0.04770776	0.5825956	0.73970004	s__Shuttleworthia_satelles
X8937656c16	-0.0169905	0.0316393	0.59346834	0.74929522	d__Bacteria; p__Firmicutes; c__Clostridia; o__Lachnospirales; f__Lachnospiraceae; g__Agathobacter
X3a107ff0183	-0.037106	0.07054564	0.6022872	0.75620504	d__Bacteria; p__Spirochaetota; c__Spirochaetia; o__Spirochaetales; f__Spirochaetaceae; g__Treponema; s__Treponema_lecithinolyticum
X5cc9206af10	0.03928244	0.07848092	0.61969503	0.76931035	d__Bacteria; p__Proteobacteria; c__Gammaproteobacteria; o__Cardiobacteriales; f__Cardiobacteriaceae; g__Cardiobacterium; s__Cardiobacterium_valvarum
c4269a6e9bd	-0.0345938	0.0703797	0.62634117	0.76931035	s__Streptococcus_mutans
X47ad35356a	0.0347717	0.06915317	0.61800045	0.76931035	d__Bacteria; p__Firmicutes; c__Bacilli; o__Staphylococcales; f__Staphylococcaceae; g__Staphylococcus
X6e086c4336	0.03402625	0.06886686	0.62423992	0.76931035	s__Bulleidia_extructa
X9886d89016	-0.0388316	0.08259552	0.6413501	0.78050593	s__uncultured_bacterium
f36c0bdcb54	0.0135171	0.02883743	0.64236329	0.78050593	s__Tannerella_sp.
faa2a7dbe2ac	0.03475809	0.07598312	0.65038926	0.78603194	s__unidentified_eubacterium
X465c45bc4b	0.03489306	0.07739761	0.65391815	0.78609309	d__Bacteria; p__Actinobacteriota; c__Actinobacteria; o__Actinomycetales; f__Actinomycetaceae; g__Actinomyces
X90b3d39699	0.03454562	0.07822448	0.66146169	0.79030483	d__Bacteria; p__Firmicutes; c__Bacilli; o__Mycoplasmatales; f__Mycoplasmataceae; g__Mycoplasma
X86c98ea884	-0.0312719	0.07144036	0.66441556	0.79030483	d__Bacteria; p__Firmicutes; c__Clostridia; o__Lachnospirales; f__Lachnospiraceae; g__Lachnoanaerobaculum
X5bb7fe5b5b	0.04333907	0.10334402	0.67761262	0.80178247	s__Tannerella_forsythia
X37ff76f02e7	-0.0403014	0.09855151	0.68541947	0.80679583	d__Bacteria; p__Proteobacteria; c__Gammaproteobacteria; o__Pasteurellales; f__Pasteurellaceae; g__Haemophilus
X70b2e89c1c	-0.0143998	0.03596981	0.69134455	0.80955372	d__Bacteria; p__Firmicutes; c__Negativicutes; o__Veillonellales-Selenomonadales; f__Veillonellaceae; g__Veillonella
ac5402de1dd	0.0385033	0.09892988	0.69960283	0.81082174	s__Bacteroides_vulgatus
dd3ff31d00fb	0.02496986	0.06354647	0.69691241	0.81082174	d__Bacteria; p__Spirochaetota; c__Spirochaetia; o__Spirochaetales; f__Spirochaetaceae; g__Treponema
b6b48b3adfb	0.01582688	0.04299424	0.71516272	0.8246264	d__Bacteria; p__Patescibacteria; c__Gracilibacteria; o__Absconditabacteriales_(SR1); f__Absconditabacteriales_(SR1); g__Absconditabacteriales_(SR1); s__SR1_bacterium
X400398fc36	0.00841513	0.02369702	0.72482678	0.83152717	d__Bacteria; p__Actinobacteriota; c__Actinobacteria; o__Bifidobacteriales; f__Bifidobacteriaceae; g__Alloscardovia; s__Alloscardovia_omnicolens
X64cafef490d	-0.0232571	0.06738315	0.73227774	0.831632	d__Bacteria; p__Proteobacteria; c__Gammaproteobacteria; o__Burkholderiales; f__Neisseriaceae
a9497011c5b	-0.040487	0.11586922	0.72868206	0.831632	d__Bacteria; p__Spirochaetota; c__Spirochaetia; o__Spirochaetales; f__Spirochaetaceae; g__Treponema; s__Treponema_socranskii
X09251debfc0	-0.0177776	0.05235949	0.7362611	0.83197504	s__Prevotella_oulorum
X185f287b8b	0.02353891	0.07280084	0.74771542	0.83273081	d__Bacteria; p__Patescibacteria; c__Saccharimonadia; o__Saccharimonadales; f__Saccharimonadaceae; g__TM7x; s__uncultured_bacterium
X5d62137a2c	-0.0221014	0.06921233	0.75151354	0.83273081	d__Bacteria; p__Firmicutes; c__Negativicutes; o__Veillonellales-Selenomonadales; f__Veillonellaceae; g__Dialister
X8b159e3b7b	-0.0304954	0.09553491	0.75166852	0.83273081	g__Mogibacterium
X7892a2e5b5	-0.0307969	0.09311329	0.74296271	0.83273081	d__Bacteria; p__Bacteroidota; c__Bacteroidia; o__Bacteroidales; f__Prevotellaceae; g__Prevotella
X2737308c23	-0.021555	0.07380495	0.77206823	0.84293439	d__Bacteria; p__Actinobacteriota; c__Actinobacteria; o__Corynebacteriales; f__Corynebacteriaceae; g__Corynebacterium; s__Corynebacterium_durum
X5b3f21fd2c0	-0.0188193	0.0642167	0.77113087	0.84293439	g__Selenomonas

X363d09a1e6	0.0067	0.02244489	0.76725369	0.84293439	d__Bacteria; p__Proteobacteria; c__Gammaproteobacteria; o__Burkholderiales; f__Neisseriaceae
a6e07e769ad	-0.0043192	0.01588019	0.7873149	0.85544792	d__Bacteria; p__Proteobacteria; c__Gammaproteobacteria; o__Burkholderiales; f__Neisseriaceae; g__Neisseria
X5ba515c461	0.021947	0.08315661	0.79342258	0.85795935	s__Treponema_denticola
e9fd215766b	0.01991054	0.08101544	0.80717911	0.86519017	d__Bacteria; p__Synergistota; c__Synergistia; o__Synergistales; f__Synergistaceae; g__Fretibacterium
e8cff5dd9314	0.02285512	0.09317035	0.80776604	0.86519017	d__Bacteria; p__Firmicutes; c__Bacilli; o__Lactobacillales
f7c8e3b65c06	0.01853291	0.07811184	0.81393262	0.86536486	d__Bacteria; p__Proteobacteria; c__Gammaproteobacteria; o__Burkholderiales; f__Burkholderiaceae; g__Lautropia
X248d9c393f	0.01403043	0.05975542	0.81558724	0.86536486	d__Bacteria; p__Bacteroidota; c__Bacteroidia; o__Bacteroidales; f__Prevotellaceae; g__Prevotella
X987a275a84	-0.0150508	0.07232544	0.83630127	0.88319667	s__unidentified
X315da8ce0c	0.00274635	0.01467432	0.85271067	0.88655555	s__Treponema_medium
X2714c3e6dd	0.00828548	0.04501864	0.85517306	0.88655555	d__Bacteria; p__Bacteroidota; c__Bacteroidia; o__Bacteroidales; f__Prevotellaceae; g__Alloprevotella
X4d861a31ad	-0.0081842	0.0414312	0.84465284	0.88655555	d__Bacteria; p__Firmicutes; c__Clostridia; o__Peptostreptococcales-Tissierellales; f__Anaerovoracaceae; g__Family_XIII_UCG-001; s__Eubacterium_sp.
a650f349bbd	-0.0082546	0.04441981	0.85371542	0.88655555	d__Bacteria; p__Firmicutes; c__Bacilli; o__Lactobacillales; f__Streptococcaceae; g__Streptococcus
X4ba52fb94b	0.01362963	0.07872004	0.86365897	0.89126451	d__Bacteria; p__Firmicutes; c__Clostridia; o__Peptostreptococcales-Tissierellales; f__Peptostreptococcaceae; g__Peptostreptococcus
X52b25decd3	-0.0117061	0.08682584	0.8935701	0.91378662	d__Bacteria; p__Firmicutes; c__Negativicutes; o__Veillonellales-Selenomonadales; f__Selenomonadaceae; g__Selenomonas; s__Selenomonas_sp.
X84d6422c40	0.00734662	0.05290415	0.89028588	0.91378662	d__Bacteria; p__Firmicutes; c__Negativicutes; o__Veillonellales-Selenomonadales; f__Selenomonadaceae
f331f41dbea9	0.00249128	0.0284017	0.93060529	0.94312465	d__Bacteria; p__Proteobacteria; c__Gammaproteobacteria; o__Burkholderiales; f__Neisseriaceae; g__Simonsiella; s__Simonsiella_muelleri
c3e85f243577	0.00463325	0.05065223	0.92768266	0.94312465	s__Lachnospiraceae_bacterium
f1da0f084f3e	-0.0033523	0.05800637	0.9542617	0.9627819	d__Bacteria; p__Proteobacteria; c__Gammaproteobacteria; o__Pasteurellales; f__Pasteurellaceae
X690754306a	-0.0034634	0.07797424	0.96485325	0.96914148	g__Campylobacter
X98e1e82c46	-0.0017225	0.05658225	0.97590335	0.97590335	s__uncultured_bacterium

* positive coefficient = increased in preservative samples

Table S2. Comparison of taxa distinguished by alternative collection methods, based on Maaslin2 analysis of absolute abundance, including features with >10% non-zero values. Features highlighted in yellow are significant

feature	coef*	stderr	pval	qval	annotation
X1a0bea5ccf9	0.63030208	0.07364918	3.24E-09	3.50E-07	d__Bacteria; p__Fusobacteriota; c__Fusobacteriia; o__Fusobacteriales; f__Fusobacteriaceae; g__Fusobacterium
c5adecb440a	0.56818558	0.06574803	2.37E-09	3.50E-07	d__Bacteria; p__Fusobacteriota; c__Fusobacteriia; o__Fusobacteriales; f__Fusobacteriaceae; g__Fusobacterium
X70dce344c1	0.51279608	0.07261227	1.09E-07	7.88E-06	d__Bacteria; p__Actinobacteriota; c__Actinobacteria; o__Corynebacteriales; f__Corynebacteriaceae; g__Corynebacterium; s__Corynebacterium_matruchotii
X9ac5fec3962	-0.2279828	0.03908454	3.29E-06	1.78E-04	d__Bacteria; p__Actinobacteriota; c__Actinobacteria; o__Actinomycetales; f__Actinomycetaceae; g__Actinomyces; s__Schaalia_odontolytica
X446810522	0.44373772	0.08381384	1.22E-05	5.26E-04	d__Bacteria; p__Bacteroidota; c__Bacteroidia; o__Bacteroidales; f__Prevotellaceae; g__Prevotella; s__Prevotella_nigrescens
ea0ff7a003ae	0.4663254	0.09320363	2.68E-05	9.65E-04	d__Bacteria; p__Bacteroidota; c__Bacteroidia; o__Bacteroidales; f__Porphyromonadaceae; g__Porphyromonas; s__unidentified
ea6265476f0	0.31709143	0.06552338	4.86E-05	1.50E-03	s__uncultured_bacterium
ebfae3fd8d9d	0.47579371	0.10163743	6.53E-05	1.76E-03	d__Bacteria; p__Bacteroidota; c__Bacteroidia; o__Bacteroidales; f__Prevotellaceae; g__Prevotella
X7be9bac1b1	-0.1385381	0.03135101	1.35E-04	0.00324675	d__Bacteria; p__Firmicutes; c__Bacilli; o__Lactobacillales; f__Streptococcaceae; g__Streptococcus
X0ae0be3085	0.41879427	0.09570756	1.63E-04	0.00326989	d__Bacteria; p__Fusobacteriota; c__Fusobacteriia; o__Fusobacteriales; f__Fusobacteriaceae; g__Fusobacterium
X82b92d7bd	-0.2118433	0.04853485	1.67E-04	0.00326989	d__Bacteria; p__Actinobacteriota; c__Actinobacteria; o__Micrococcales; f__Micrococcaceae; g__Rothia
X4c6bfea158	0.30640099	0.07368051	2.81E-04	0.0050496	d__Bacteria; p__Bacteroidota; c__Bacteroidia; o__Bacteroidales; f__Prevotellaceae; g__Prevotella; s__Prevotella_melaninogenica
ce34506b9e1	0.18019612	0.0461226	5.35E-04	0.00769388	d__Bacteria; p__Campilobacterota; c__Campylobacteria; o__Campylobacterales; f__Campylobacteraceae; g__Campylobacter; s__unidentified
a8a8f3c4482	0.17118852	0.04326124	4.76E-04	0.00769388	d__Bacteria; p__Fusobacteriota; c__Fusobacteriia; o__Fusobacteriales; f__Fusobacteriaceae; g__Fusobacterium
cce0f21cdf55	-0.2216909	0.05705291	5.70E-04	0.00769388	d__Bacteria; p__Actinobacteriota; c__Actinobacteria; o__Micrococcales; f__Micrococcaceae; g__Rothia; s__Rothia_mucilaginosa
X0540bf08e9	-0.2567905	0.06556985	5.21E-04	0.00769388	d__Bacteria; p__Actinobacteriota; c__Actinobacteria; o__Actinomycetales; f__Actinomycetaceae; g__Actinomyces; s__Actinomyces_graevenitzii
fae8f7a4814d	0.30719935	0.08092712	7.44E-04	0.00945066	d__Bacteria; p__Bacteroidota; c__Bacteroidia; o__Bacteroidales; f__Prevotellaceae; g__Prevotella; s__Prevotella_pallens
X6a2914e072	0.23949425	0.06493345	9.60E-04	0.01151514	d__Bacteria; p__Bacteroidota; c__Bacteroidia; o__Bacteroidales; f__Prevotellaceae; g__Prevotella; s__Prevotella_sp.
X9d4e16844	-0.1827913	0.04977756	1.05E-03	0.01190987	d__Bacteria; p__Firmicutes; c__Bacilli; o__Staphylococcales; f__Gemellaceae; g__Gemella
X3bce60a047	0.28033934	0.07803662	0.00120211	0.01298276	d__Bacteria; p__Bacteroidota; c__Bacteroidia; o__Flavobacteriales; f__Flavobacteriaceae; g__Capnocytophaga
X8903ee9324	0.31590626	0.0891876	0.0013935	0.01340662	d__Bacteria; p__Bacteroidota; c__Bacteroidia; o__Bacteroidales; f__Prevotellaceae; g__Prevotella; s__Prevotella_nanceiensis
f6852cab4e2	0.24052209	0.06671064	0.00132182	0.01340662	d__Bacteria; p__Bacteroidota; c__Bacteroidia; o__Bacteroidales; f__Prevotellaceae; g__Prevotella; s__Prevotella_veroralis
X3cc657dfb4	0.33919118	0.09566251	0.00142756	0.01340662	d__Bacteria; p__Bacteroidota; c__Bacteroidia; o__Flavobacteriales; f__Flavobacteriaceae; g__Capnocytophaga
X7b9a7ec76b	0.24242587	0.06892839	0.00156148	0.01405332	d__Bacteria; p__Bacteroidota; c__Bacteroidia; o__Flavobacteriales; f__Weeksellaceae; g__Bergeyella
X37b947daa0	0.27143305	0.08089602	0.00222976	0.01926513	d__Bacteria; p__Fusobacteriota; c__Fusobacteriia; o__Fusobacteriales; f__Leptotrichiaceae; g__Leptotrichia; s__Leptotrichia_hongkongensis
X8bbc849c70	0.15659013	0.04679216	0.00248866	0.02067502	d__Bacteria; p__Fusobacteriota; c__Fusobacteriia; o__Fusobacteriales; f__Fusobacteriaceae; g__Fusobacterium
aa5f922ae53	0.21878784	0.06727284	0.0031464	0.02517118	d__Bacteria; p__Fusobacteriota; c__Fusobacteriia; o__Fusobacteriales; f__Leptotrichiaceae; g__Leptotrichia; s__Leptotrichia_shahii
eb3043d340	0.23996993	0.07564607	0.00369252	0.02848515	d__Bacteria; p__Bacteroidota; c__Bacteroidia; o__Bacteroidales; f__Prevotellaceae; g__Prevotella; s__Prevotella_denticola
b3d06445a91	-0.1121181	0.03555318	0.00388474	0.02893459	d__Bacteria; p__Firmicutes; c__Bacilli; o__Lactobacillales
X13d195efab	0.12363809	0.03966097	0.00409738	0.02950112	d__Bacteria; p__Firmicutes; c__Bacilli; o__RF39; f__RF39; g__RF39; s__uncultured_bacterium
X39f89a63c1	0.2518566	0.08259733	0.00476702	0.03227664	d__Bacteria; p__Bacteroidota; c__Bacteroidia; o__Bacteroidales; f__Porphyromonadaceae; g__Porphyromonas; s__Porphyromonas_catoniae
X3ee072a59b	-0.1521727	0.0498748	0.00493115	0.03227664	d__Bacteria; p__Actinobacteriota; c__Actinobacteria; o__Micrococcales; f__Micrococcaceae; g__Rothia
X39d23124f2	0.16202994	0.05295407	0.00490696	0.03227664	d__Bacteria; p__Fusobacteriota; c__Fusobacteriia; o__Fusobacteriales; f__Leptotrichiaceae; g__Leptotrichia; s__Leptotrichia_wadei
X31b250015	0.27210346	0.08974088	0.00535105	0.03399493	d__Bacteria; p__Firmicutes; c__Clostridia; o__Peptococcales; f__Peptococcaceae; g__Peptococcus
X10953969f0	0.16929114	0.05790881	0.00631596	0.03789573	d__Bacteria; p__Firmicutes; c__Clostridia; o__Lachnospirales; f__Lachnospiraceae; g__Catonella
a159622ef88	0.32965064	0.11105178	0.00615857	0.03789573	d__Bacteria; p__Firmicutes; c__Clostridia; o__Clostridia_UCG-014; f__Clostridia_UCG-014; g__Clostridia_UCG-014; s__Clostridiales_bacterium
fe87c08549ff	0.12730581	0.04357148	0.00693261	0.03919199	d__Bacteria; p__Bacteroidota; c__Bacteroidia; o__Bacteroidales; f__Prevotellaceae; g__Prevotella; s__Prevotella_melaninogenica
ce33f26f3bd4	0.16814724	0.05786543	0.00708387	0.03919199	d__Bacteria; p__Fusobacteriota; c__Fusobacteriia; o__Fusobacteriales; f__Leptotrichiaceae; g__Leptotrichia
d4dc4e8d0ff0	0.27187183	0.09349059	0.00725778	0.03919199	d__Bacteria; p__Firmicutes; c__Clostridia; o__Lachnospirales; f__Lachnospiraceae; g__Catonella; s__uncultured_bacterium
X8005a5fccd	0.14643601	0.05212754	0.00721259	0.03919199	d__Bacteria; p__Fusobacteriota; c__Fusobacteriia; o__Fusobacteriales; f__Leptotrichiaceae; g__Leptotrichia; s__Leptotrichia_goodfellowii
X5e947f6ca8	0.19269618	0.06713666	0.00751785	0.03960621	d__Bacteria; p__Fusobacteriota; c__Fusobacteriia; o__Fusobacteriales; f__Leptotrichiaceae; g__Leptotrichia
X72cbe4cdd9	0.33503998	0.11774637	0.00844301	0.04240839	d__Bacteria; p__Bacteroidota; c__Bacteroidia; o__Bacteroidales; f__Prevotellaceae; g__Alloprevotella; s__Alloprevotella_rava
fe02f7b94a8e	0.18057783	0.06388649	0.00848321	0.04240839	s__Leptotrichia_hofstadii

X6362083e09	0.25749346	0.09098913	0.00863875	0.04240839	d__Bacteria; p__Proteobacteria; c__Gammaproteobacteria; o__Cardiobacteriales; f__Cardiobacteriaceae; g__Cardiobacterium; s__uncultured_bacterium
X6a66cd42df	0.20307553	0.07225151	0.00947802	0.04540918	d__Bacteria; p__Fusobacteriota; c__Fusobacteriia; o__Fusobacteriales; f__Leptotrichiaceae; g__Leptotrichia; s__Leptotrichia_sp.
e192c41a11d	0.18667621	0.06711215	0.00967047	0.04540918	d__Bacteria; p__Fusobacteriota; c__Fusobacteriia; o__Fusobacteriales; f__Leptotrichiaceae; g__Leptotrichia; s__Leptotrichia_sp.
cbe5861699e	0.15552613	0.05922192	0.01353751	0.06221495	d__Bacteria; p__Bacteroidota; c__Bacteroidia; o__Bacteroidales; f__Prevotellaceae; g__Prevotella; s__Prevotella_pleuritidis
X64bdd6a571	0.13739915	0.05358597	0.01632627	0.06781683	d__Bacteria; p__Bacteroidota; c__Bacteroidia; o__Bacteroidales; f__Prevotellaceae; g__Prevotella; s__Prevotella_intermedia
X602510f333	0.16354621	0.06331677	0.01541566	0.06781683	d__Bacteria; p__Bacteroidota; c__Bacteroidia; o__Bacteroidales; f__Prevotellaceae; g__Prevotella; s__Prevotella_melaninogenica
X6e70d256d3	0.18107178	0.07080937	0.01625129	0.06781683	d__Bacteria; p__Bacteroidota; c__Bacteroidia; o__Bacteroidales; f__Prevotellaceae; g__Alloprevotella; s__uncultured_Bacteroidetes
X1fdb57d940	0.15253817	0.05882346	0.01514486	0.06781683	d__Bacteria; p__Bacteroidota; c__Bacteroidia; o__Bacteroidales; f__Porphyromonadaceae; g__Porphyromonas; s__uncultured_bacterium
X0eb193966e	0.24360284	0.0948507	0.01610644	0.06781683	d__Bacteria; p__Patescibacteria; c__Gracilibacteria; o__Absconditabacteriales_(SR1); f__Absconditabacteriales_(SR1); g__Absconditabacteriales_(SR1); s__SR1_bacterium
df8284858d8	0.14910674	0.06008837	0.01952818	0.07811272	d__Bacteria; p__Bacteroidota; c__Bacteroidia; o__Bacteroidales; f__Prevotellaceae; g__Prevotella; s__Prevotella_oris
aadada17f552	0.13937695	0.05609768	0.01928471	0.07811272	d__Bacteria; p__Firmicutes; c__Clostridia; o__Lachnospirales; f__Lachnospiraceae; g__Butyrivibrio
X685b8609f6	0.19501501	0.0799573	0.02130186	0.08365822	d__Bacteria; p__Bacteroidota; c__Bacteroidia; o__Bacteroidales; f__Prevotellaceae; g__Prevotella; s__Prevotella_shahii
b29d6e91ae4	0.18851379	0.07772269	0.02184478	0.08425845	d__Bacteria; p__Proteobacteria; c__Gammaproteobacteria; o__Burkholderiales; f__Neisseriaceae
a6369fc8915	0.32351666	0.13456716	0.02285719	0.08661671	d__Bacteria; p__Bacteroidota; c__Bacteroidia; o__Bacteroidales; f__Porphyromonadaceae; g__Porphyromonas
X9af90fa49fa	0.3071051	0.12801523	0.0233673	0.08702304	d__Bacteria; p__Bacteroidota; c__Bacteroidia; o__Bacteroidales; f__Prevotellaceae; g__Prevotella; s__Prevotella_nanceiensis
cf4e43ffaa77b	0.16902072	0.0714805	0.02532073	0.09269962	d__Bacteria; p__Firmicutes; c__Clostridia; o__Peptostreptococcales-Tissierellales; f__Peptostreptococcaceae; g__Filifactor; s__Filifactor_alocis
X6c83fec6e8	-0.1253294	0.0540616	0.02794977	0.10061916	d__Bacteria; p__Firmicutes; c__Negativicutes; o__Veillonellales-Selenomonadales; f__Veillonellaceae; g__Veillonella
af684f96f945	0.21192675	0.09318854	0.03040994	0.10768111	d__Bacteria; p__Bacteroidota; c__Bacteroidia; o__Bacteroidales; f__Prevotellaceae; g__Alloprevotella; s__Alloprevotella_tannerae
X1f4c8ba04b	-0.1150381	0.05068438	0.03134278	0.1091942	d__Bacteria; p__Actinobacteriota; c__Actinobacteria; o__Actinomycetales; f__Actinomycetaceae; g__Actinomyces; s__Schaalia_odontolytica
X0a69c0ff3a3	0.2430214	0.10865172	0.03319244	0.11357506	d__Bacteria; p__Firmicutes; c__Clostridia; o__Clostridia_UCG-014; f__Clostridia_UCG-014; g__Clostridia_UCG-014
X76075eede2	0.10628636	0.04753845	0.03365187	0.11357506	d__Bacteria; p__Proteobacteria; c__Gammaproteobacteria; o__Pasteurellales; f__Pasteurellaceae; g__Aggregatibacter
X29e53aaab0	-0.1841715	0.08274156	0.03447886	0.11380791	d__Bacteria; p__Actinobacteriota; c__Actinobacteria; o__Micrococcales; f__Micrococcaceae; g__Rothia
X252d0fd660	0.18875679	0.0848138	0.03477464	0.11380791	s__Scardovia_wiggsiae
X219d2ac2e4	0.1776859	0.08392789	0.04305582	0.13676555	d__Bacteria; p__Firmicutes; c__Clostridia; o__Lachnospirales; f__Lachnospiraceae; g__Lachnoanaerobaculum
d4c49968a5f	0.06337838	0.02989133	0.0430157	0.13676555	d__Bacteria; p__Firmicutes; c__Clostridia; o__Peptostreptococcales-Tissierellales; f__Peptostreptococcaceae; g__Peptoanaerobacter; s__Peptoanaerobacter_stomatis
X7d4bc1c15b	-0.1376067	0.06629372	0.04738614	0.14833923	d__Bacteria; p__Firmicutes; c__Bacilli; o__Lactobacillales; f__Streptococcaceae; g__Streptococcus
X49b6d0b43a	0.13104142	0.06386418	0.0499394	0.15409871	d__Bacteria; p__Bacteroidota; c__Bacteroidia; o__Bacteroidales; f__Prevotellaceae; g__Prevotella; s__Prevotella_intermedia
X3a22f9a8f97	0.15582304	0.07635599	0.05104386	0.15528835	d__Bacteria; p__Bacteroidota; c__Bacteroidia; o__Bacteroidales; f__Prevotellaceae; g__Alloprevotella; s__Alloprevotella_tannerae
X5ba90da636	0.17750306	0.08709026	0.05232958	0.15698874	d__Bacteria; p__Fusobacteriota; c__Fusobacteriia; o__Fusobacteriales; f__Leptotrichiaceae; g__Leptotrichia
f83bc32e60e9	-0.0373075	0.01876128	0.05663077	0.16756502	d__Bacteria; p__Actinobacteriota; c__Actinobacteria; o__Bifidobacteriales; f__Bifidobacteriaceae; g__Bifidobacterium
ffa12a95bac2	0.17937392	0.09184213	0.06087628	0.17769294	d__Bacteria; p__Bacteroidota; c__Bacteroidia; o__Bacteroidales; f__Prevotellaceae; g__Prevotella; s__Prevotella_pallens
c4007722892	0.12926331	0.06663617	0.06198496	0.17851668	d__Bacteria; p__Bacteroidota; c__Bacteroidia; o__Bacteroidales; f__Prevotellaceae; g__Prevotella; s__Prevotella_pleuritidis
d9b76ee51ff2	-0.1124386	0.05803035	0.06293937	0.1788803	d__Bacteria; p__Firmicutes; c__Bacilli; o__Lactobacillales; f__Streptococcaceae; g__Streptococcus
X0c2876c22b	0.15318443	0.07927844	0.06410443	0.17982542	s__Leptotrichia_buccalis
X4917691360	0.12677648	0.066402	0.06610518	0.18306051	d__Bacteria; p__Fusobacteriota; c__Fusobacteriia; o__Fusobacteriales; f__Leptotrichiaceae; g__Leptotrichia; s__Leptotrichia_sp.
X601dcc5af9	0.14301146	0.07628232	0.07135736	0.19510366	d__Bacteria; p__Firmicutes; c__Negativicutes; o__Veillonellales-Selenomonadales; f__Selenomonadaceae
f3cac486ad34	0.10452105	0.05596831	0.07269422	0.19627441	d__Bacteria; p__Bacteroidota; c__Bacteroidia; o__Bacteroidales; f__Prevotellaceae; g__Prevotella; s__Prevotella_buccae
X2fd80b8b34	0.16120601	0.08818605	0.0786784	0.20231588	d__Bacteria; p__Bacteroidota; c__Bacteroidia; o__Bacteroidales; f__Prevotellaceae; g__Prevotella; s__Prevotella_genomosp.
f757847365a	0.16138226	0.08851394	0.07821019	0.20231588	d__Bacteria; p__Bacteroidota; c__Bacteroidia; o__Bacteroidales; f__Tannerellaceae; g__Tannerella
e796676dcb0	0.08327362	0.04517596	0.07709849	0.20231588	d__Bacteria; p__Actinobacteriota; c__Coriobacteriia; o__Coriobacteriales; f__Atopobiaceae; g__Atopobium
X1bb5836797	0.18432913	0.10084176	0.07840049	0.20231588	d__Bacteria; p__Firmicutes; c__Negativicutes; o__Veillonellales-Selenomonadales; f__Selenomonadaceae; g__Selenomonas; s__Selenomonas_sputigena
e2c3ff4f6471	0.05463055	0.03011955	0.08085077	0.20545607	d__Bacteria; p__Actinobacteriota; c__Actinobacteria; o__Bifidobacteriales; f__Bifidobacteriaceae; g__Bifidobacterium
X502ecbbaeb	0.0918531	0.05071946	0.08323451	0.20866522	d__Bacteria; p__Bacteroidota; c__Bacteroidia; o__Bacteroidales; f__Prevotellaceae; g__Prevotella; s__Prevotella_oralis
X6c1f3bf3e21	0.18489195	0.10315799	0.08431374	0.20866522	d__Bacteria; p__Firmicutes; c__Clostridia; o__Peptostreptococcales-Tissierellales; f__Peptostreptococcales-Tissierellales; g__Parvimonas
X1986030e3c	0.143091	0.08028872	0.08501176	0.20866522	d__Bacteria; p__Proteobacteria; c__Gammaproteobacteria; o__Pasteurellales; f__Pasteurellaceae; g__Haemophilus

X9e53a677b1	0.18831734	0.1063924	0.08885954	0.21524631	d__Bacteria; p__Patescibacteria; c__Gracilibacteria; o__Absconditabacteriales_(SR1); f__Absconditabacteriales_(SR1); g__Absconditabacteriales_(SR1); s__SR1_bacterium
X15dd83460(	0.14390477	0.08152009	0.08968596	0.21524631	d__Bacteria; p__Proteobacteria; c__Gammaproteobacteria; o__Pasteurellales; f__Pasteurellaceae; g__Aggregatibacter
cca22a273fa1	-0.1119905	0.06472754	0.09457016	0.22447423	d__Bacteria; p__Firmicutes; c__Negativicutes; o__Veillonellales-Selenomonadales; f__Veillonellaceae; g__Megasphaera; s__Megasphaera_micronuciformis
X44598e75a1	0.13668605	0.08028549	0.09954079	0.2311915	d__Bacteria; p__Bacteroidota; c__Bacteroidia; o__Sphingobacteriales; f__Lentimicrobiaceae; g__Lentimicrobium; s__unidentified
X10a9a08874	-0.0790263	0.04628257	0.09881301	0.2311915	d__Bacteria; p__Proteobacteria; c__Gammaproteobacteria; o__Pasteurellales; f__Pasteurellaceae; g__Actinobacillus
da19337673b	0.09086119	0.05416769	0.10441523	0.23993287	d__Bacteria; p__Firmicutes; c__Clostridia; o__Lachnospirales; f__Lachnospiraceae; g__Johnsonella; s__uncultured_bacterium
X4458477e8a	0.12294631	0.07367295	0.10662609	0.24243405	d__Bacteria; p__Bacteroidota; c__Bacteroidia; o__Bacteroidales; f__Prevotellaceae; g__Prevotella; s__Prevotella_oris
b7a041d2927	0.11908978	0.07207401	0.11026295	0.24252608	d__Bacteria; p__Campilobacterota; c__Campylobacteria; o__Campylobacterales; f__Campylobacteraceae; g__Campylobacter
ce4de2da787	0.09828379	0.05961865	0.11072094	0.24252608	d__Bacteria; p__Bacteroidota; c__Bacteroidia; o__Bacteroidales; f__Prevotellaceae; g__Prevotella; s__Prevotella_oulorum
X47c98abffb7	-0.1681642	0.10215325	0.11228059	0.24252608	d__Bacteria; p__Firmicutes; c__Bacilli; o__Lactobacillales; f__Streptococcaceae; g__Streptococcus; s__Streptococcus_anginosus
ffb88213324l	0.09038044	0.05448897	0.1085	0.24252608	d__Bacteria; p__Firmicutes; c__Bacilli; o__RF39; f__RF39; g__RF39; s__Firmicutes_oral
X68db566a60	-0.1253035	0.07631778	0.1121153	0.24252608	d__Bacteria; p__Actinobacteriota; c__Actinobacteria; o__Actinomycetales; f__Actinomycetaceae; g__Actinomyces
X6fd5fcf5367	0.2051158	0.12614925	0.11516119	0.24628532	d__Bacteria; p__Bacteroidota; c__Bacteroidia; o__Bacteroidales; f__Paludibacteraceae; g__F0058; s__uncultured_bacterium
X98e1e82c46	0.05762923	0.03569435	0.11836649	0.25065845	d__Bacteria; p__Bacteroidota; c__Bacteroidia; o__Flavobacteriales; f__Weeksellaceae; g__Bergeyella; s__uncultured_bacterium
b8c6906ea99	-0.1026129	0.06409096	0.12099784	0.25374303	d__Bacteria; p__Actinobacteriota; c__Actinobacteria; o__Micrococcales; f__Micrococcaceae; g__Rothia
X7217df85b1	0.20028827	0.1257673	0.12247075	0.25436234	d__Bacteria; p__Bacteroidota; c__Bacteroidia; o__Bacteroidales; f__Prevotellaceae; g__Alloprevotella; s__unidentified
X2c1a555f04(	0.04171383	0.02691147	0.13256521	0.27270558	d__Bacteria; p__Campilobacterota; c__Campylobacteria; o__Campylobacterales; f__Campylobacteraceae; g__Campylobacter
X499137328(	0.08827246	0.05772782	0.13886947	0.2829793	d__Bacteria; p__Firmicutes; c__Clostridia; o__Lachnospirales; f__Lachnospiraceae; g__Oribacterium
d44d4b8a0a3	0.05975536	0.03973491	0.14425337	0.29120307	d__Bacteria; p__Proteobacteria; c__Gammaproteobacteria; o__Burkholderiales; f__Neisseriaceae; g__Neisseria; s__Neisseria_oralis
aacaf5f360c1	-0.1176584	0.08302548	0.167572	0.335144	d__Bacteria; p__Firmicutes; c__Negativicutes; o__Veillonellales-Selenomonadales; f__Selenomonadaceae; g__Selenomonas; s__Selenomonas_artemidis
X19daa442e7	0.02941471	0.02089707	0.17018611	0.33724953	d__Bacteria; p__Firmicutes; c__Negativicutes; o__Veillonellales-Selenomonadales; f__Veillonellaceae
f434ebfe13e3	0.08513905	0.06102656	0.17400441	0.34168138	s__Veillonella_sp.
X3827b3842a	-0.1267568	0.0916861	0.17805474	0.3464849	d__Bacteria; p__Actinobacteriota; c__Actinobacteria; o__Actinomycetales; f__Actinomycetaceae; g__Actinomyces
X641e0a0923	0.05913586	0.04343519	0.18450295	0.35582712	d__Bacteria; p__Firmicutes; c__Bacilli; o__Lactobacillales; f__Streptococcaceae; g__Streptococcus; s__Streptococcus_anginosus
X4ba52fb94b	0.0780882	0.05974199	0.20178678	0.38571632	g__Peptostreptococcus
d79a701e5a4	0.09152209	0.07091336	0.2078827	0.39153815	d__Bacteria; p__Bacteroidota; c__Bacteroidia; o__Bacteroidales; f__Prevotellaceae; g__Prevotella; s__Prevotella_saccharolytica
X0755e2d58b	0.13077793	0.10153682	0.20845781	0.39153815	d__Bacteria; p__Proteobacteria; c__Gammaproteobacteria; o__Burkholderiales; f__Neisseriaceae; g__Kingella; s__uncultured_bacterium
X38e3b7abf4(	0.07233874	0.05699282	0.21591329	0.40204544	d__Bacteria; p__Fusobacteriota; c__Fusobacteriia; o__Fusobacteriales; f__Fusobacteriaceae; g__Fusobacterium
fdcd6808ef82	0.12882638	0.10270919	0.21985306	0.40588258	d__Bacteria; p__Proteobacteria; c__Gammaproteobacteria; o__Burkholderiales; f__Neisseriaceae; g__Neisseria
X447ee7f88c(	0.04762797	0.03817071	0.2222275	0.40678931	d__Bacteria; p__Fusobacteriota; c__Fusobacteriia; o__Fusobacteriales; f__Fusobacteriaceae; g__Fusobacterium
b4f5c5ff1599	0.06939245	0.05589503	0.2252653	0.40888491	d__Bacteria; p__Firmicutes; c__Negativicutes; o__Veillonellales-Selenomonadales; f__Selenomonadaceae; g__Selenomonas
X510f6fbe9dc	0.08343774	0.0678267	0.22918914	0.40913102	d__Bacteria; p__Firmicutes; c__Clostridia; o__Peptostreptococcales-Tissierellales; f__Anaerovoracaceae; g__[Eubacterium]_brachy_group; s__Eubacterium_brachy
X5a0df24ab3(	-0.057465	0.04651549	0.22795358	0.40913102	s__uncultured_bacterium
X984ef97167	0.05839188	0.04799666	0.23450702	0.41519275	d__Bacteria; p__Firmicutes; c__Clostridia; o__Lachnospirales; f__Lachnospiraceae; g__Oribacterium; s__Oribacterium_parvum
X4904c5ac70	0.04994956	0.04209748	0.24404046	0.42751891	d__Bacteria; p__Bacteroidota; c__Bacteroidia; o__Flavobacteriales; f__Flavobacteriaceae; g__Capnocytophaga
ae92f7ff4fd72	0.12408566	0.10452505	0.24542752	0.42751891	d__Bacteria; p__Firmicutes; c__Clostridia; o__Lachnospirales; f__Lachnospiraceae; g__Stomatobaculum; s__Eubacterium_sp.
X26800fe52e	0.22101962	0.18717266	0.24760307	0.4278581	d__Bacteria; p__Bacteroidota; c__Bacteroidia; o__Bacteroidales; f__Prevotellaceae; g__Alloprevotella; s__uncultured_Bacteroidetes
X78e469b5fd	-0.0413429	0.03557407	0.25524838	0.43756865	d__Bacteria; p__Firmicutes; c__Clostridia; o__Lachnospirales; f__Lachnospiraceae; g__Oribacterium
X1c138843b8	0.0678307	0.05990899	0.26799339	0.45510552	d__Bacteria; p__Bacteroidota; c__Bacteroidia; o__Flavobacteriales; f__Flavobacteriaceae; g__Capnocytophaga; s__uncultured_Capnocytophaga
f331f41dbea9	0.0264212	0.02346539	0.26969216	0.45510552	d__Bacteria; p__Proteobacteria; c__Gammaproteobacteria; o__Burkholderiales; f__Neisseriaceae; g__Simonsiella; s__Simonsiella_muelleri
X49757d4da8	0.04073471	0.03647628	0.27406064	0.45889223	d__Bacteria; p__Proteobacteria; c__Gammaproteobacteria; o__Burkholderiales; f__Neisseriaceae; g__Alysiella
f9d1dd7ca23	0.06001241	0.05545432	0.28843605	0.47558921	d__Bacteria; p__Bacteroidota; c__Bacteroidia; o__Bacteroidales; f__Prevotellaceae; g__Prevotella; s__Prevotella_shahii
X84d6422c4(	-0.0431682	0.03968051	0.2863181	0.47558921	d__Bacteria; p__Firmicutes; c__Negativicutes; o__Veillonellales-Selenomonadales; f__Selenomonadaceae
X9d863926e2	0.09414184	0.08823431	0.29555865	0.48137139	d__Bacteria; p__Proteobacteria; c__Gammaproteobacteria; o__Burkholderiales; f__Comamonadaceae; g__Comamonas; s__Ottowia_sp.
def8acc0fc88	-0.066254	0.06215882	0.29639998	0.48137139	d__Bacteria; p__Firmicutes; c__Bacilli; o__Erysipelotrichales; f__Erysipelotrichaceae; g__Solobacterium; s__Solobacterium_moorei
X41a360fde2	0.06037581	0.05782715	0.30168118	0.48268988	d__Bacteria; p__Firmicutes; c__Bacilli; o__Mycoplasmatales; f__Mycoplasmataceae; g__Mycoplasma; s__Mycoplasmopsis_arginini

b6a0f2aee9b3	0.0657426	0.06231367	0.29948818	0.48268988	d__Bacteria; p__Fusobacteriota; c__Fusobacteriia; o__Fusobacteriales; f__Leptotrichiaceae; g__Leptotrichia
X5bb7fe5b5b	0.10987056	0.10511956	0.30557965	0.48533239	d__Bacteria; p__Bacteroidota; c__Bacteroidia; o__Bacteroidales; f__Tannerellaceae; g__Tannerella; s__Tannerella_forsythia
X95348b8c18	0.06376208	0.06252726	0.31681585	0.49950528	d__Bacteria; p__Firmicutes; c__Negativicutes; o__Veillonellales-Selenomonadales; f__Veillonellaceae; g__Veillonella
X0d672b487f	0.09534966	0.09471434	0.32374882	0.50673728	d__Bacteria; p__Bacteroidota; c__Bacteroidia; o__Bacteroidales; f__Prevotellaceae; g__Prevotella; s__Prevotella_sp.
X1d8aede440	0.04023482	0.04045475	0.32964418	0.51001672	d__Bacteria; p__Firmicutes; c__Clostridia; o__Lachnospirales; f__Lachnospiraceae; g__Butyrivibrio; s__Firmicutes_oral
f36c0bdcb54	0.02718036	0.02744771	0.33056639	0.51001672	d__Bacteria; p__Bacteroidota; c__Bacteroidia; o__Bacteroidales; f__Tannerellaceae; g__Tannerella; s__Tannerella_sp.
X5b1b3a87b6	0.06254291	0.06478284	0.34148184	0.52312111	d__Bacteria; p__Spirochaetota; c__Spirochaetia; o__Spirochaetales; f__Spirochaetaceae; g__Treponema; s__Treponema_socranskii
e0f64c7eb3cc	-0.0563174	0.05927277	0.34980406	0.5303411	d__Bacteria; p__Firmicutes; c__Clostridia; o__Peptostreptococcales-Tissierellales; f__Anaerovoracaceae; g__[Eubacterium]_nodatum_group; s__[Eubacterium]_nodatum
X9e8e56f06c0	-0.0800818	0.08430521	0.35110545	0.5303411	d__Bacteria; p__Actinobacteriota; c__Actinobacteria; o__Actinomycetales; f__Actinomycetaceae; g__Actinomyces
X3a107ff0183	-0.072774	0.07774796	0.35719133	0.535787	d__Bacteria; p__Spirochaetota; c__Spirochaetia; o__Spirochaetales; f__Spirochaetaceae; g__Treponema; s__Treponema_lecithinolyticum
X2731574ed2	0.04185714	0.04576382	0.3689392	0.54959218	d__Bacteria; p__Bacteroidota; c__Bacteroidia; o__Bacteroidales; f__Prevotellaceae; g__Prevotella; s__Prevotella_maculosa
f64be8a0bee6	-0.0135257	0.01526876	0.38331287	0.56709301	d__Bacteria; p__Firmicutes; c__Negativicutes; o__Veillonellales-Selenomonadales; f__Veillonellaceae; g__Veillonella
X2f297c39eca	0.10373835	0.11795988	0.38652018	0.56794803	d__Bacteria; p__Firmicutes; c__Clostridia; o__Peptostreptococcales-Tissierellales; f__Peptostreptococcales-Tissierellales; g__Parvimonas
f1dfcb23cf4d	0.03435552	0.03993414	0.39725777	0.57286067	d__Bacteria; p__Synergistota; c__Synergistia; o__Synergistales; f__Synergistaceae; g__Fretibacterium
f1da0f084f3e	0.03068441	0.03563821	0.3969639	0.57286067	d__Bacteria; p__Proteobacteria; c__Gammaproteobacteria; o__Pasteurellales; f__Pasteurellaceae
X33206a2306	-0.0397797	0.04624544	0.39783533	0.57286067	d__Bacteria; p__Proteobacteria; c__Gammaproteobacteria; o__Pasteurellales; f__Pasteurellaceae; g__Actinobacillus; s__Haemophilus_parahaemolyticus
X82d2a5f97a	0.07543061	0.0882938	0.40047204	0.57286067	d__Bacteria; p__Bacteroidota; c__Bacteroidia; o__Bacteroidales; f__Prevotellaceae; g__Prevotella; s__Prevotella_salivae
d2e36e4099b	0.04991232	0.05931703	0.40814801	0.57788971	d__Bacteria; p__Patescibacteria; c__Saccharimonadia; o__Saccharimonadales; f__Saccharimonadaceae; g__Candidatus_Saccharimonas; s__uncultured_bacterium
ea322a31ace	0.05776785	0.06904636	0.40933854	0.57788971	d__Bacteria; p__Firmicutes; c__Clostridia; o__Peptostreptococcales-Tissierellales; f__Peptostreptococcaceae; g__[Eubacterium]_yurii_group; s__uncultured_bacterium
X690754306a	0.05447731	0.06660589	0.420369	0.58204939	d__Bacteria; p__Campilobacterota; c__Campylobacteria; o__Campylobacterales; f__Campylobacteraceae; g__Campylobacter
X3e3cbcd987	0.02625783	0.03193744	0.41815764	0.58204939	d__Bacteria; p__Bacteroidota; c__Bacteroidia; o__Bacteroidales; f__Prevotellaceae; g__Prevotella; s__Prevotella_aurantiaca
X25def15fb42	-0.0805703	0.09798667	0.41703655	0.58204939	d__Bacteria; p__Verrucomicrobiota; c__Verrucomicrobiae; o__Verrucomicrobiales; f__Akkermansiaceae; g__Akkermansia
f55030f7203	0.04628562	0.05833658	0.4315092	0.5936687	d__Bacteria; p__Bacteroidota; c__Bacteroidia; o__Bacteroidales; f__Prevotellaceae; g__Alloprevotella; s__uncultured_eubacterium
X86c98ea884	-0.0435625	0.05577757	0.44167966	0.60381523	d__Bacteria; p__Firmicutes; c__Clostridia; o__Lachnospirales; f__Lachnospiraceae; g__Lachnoanaerobaculum
fe30c2d2ad2	0.03887632	0.05238237	0.46418199	0.63058686	d__Bacteria; p__Bacteroidota; c__Bacteroidia
c4269a6e9bd	-0.0557188	0.0773366	0.47737861	0.64045826	d__Bacteria; p__Firmicutes; c__Bacilli; o__Lactobacillales; f__Streptococcaceae; g__Streptococcus; s__Streptococcus_mutans
X93233cf6a3	-0.0714469	0.09896858	0.47554266	0.64045826	d__Bacteria; p__Firmicutes; c__Bacilli; o__Lactobacillales; f__Streptococcaceae; g__Streptococcus
X643cef76cf1	0.03433559	0.04892685	0.48914574	0.65219432	d__Bacteria; p__Proteobacteria; c__Gammaproteobacteria; o__Burkholderiales; f__Neisseriaceae
X95f77f9e6df	0.04171899	0.06064186	0.49776792	0.65961884	d__Bacteria; p__Firmicutes; c__Negativicutes; o__Veillonellales-Selenomonadales; f__Veillonellaceae
X5e6aa5e331	-0.0457419	0.06795111	0.50526025	0.66546472	d__Bacteria; p__Firmicutes; c__Clostridia; o__Lachnospirales; f__Lachnospiraceae
X5ba515c461	0.05065939	0.07599363	0.51060503	0.6684284	d__Bacteria; p__Spirochaetota; c__Spirochaetia; o__Spirochaetales; f__Spirochaetaceae; g__Treponema; s__Treponema_denticola
X5cc9206af10	-0.0426627	0.06585085	0.52199283	0.6754687	d__Bacteria; p__Proteobacteria; c__Gammaproteobacteria; o__Cardiobacteriales; f__Cardiobacteriaceae; g__Cardiobacterium; s__Cardiobacterium_valvarum
X29bb5fa7f45	0.05957774	0.09295338	0.52679566	0.6754687	d__Bacteria; p__Firmicutes; c__Negativicutes; o__Veillonellales-Selenomonadales; f__Veillonellaceae; g__Veillonella
X5b3f21fd2c0	0.03712214	0.05822634	0.52906811	0.6754687	d__Bacteria; p__Firmicutes; c__Negativicutes; o__Veillonellales-Selenomonadales; f__Selenomonadaceae; g__Selenomonas
de8a1c0d12e	0.0250167	0.03864752	0.52243123	0.6754687	d__Bacteria; p__Bacteroidota; c__Bacteroidia; o__Flavobacteriales; f__Flavobacteriaceae; g__Capnocytophaga; s__uncultured_Capnocytophaga
ebd6c1d23c5	0.03012678	0.04755887	0.53161888	0.6754687	d__Bacteria; p__Proteobacteria; c__Gammaproteobacteria; o__Burkholderiales; f__Neisseriaceae; g__Neisseria
X70b2e89c1c	-0.0148287	0.02478715	0.5545691	0.69249974	d__Bacteria; p__Firmicutes; c__Negativicutes; o__Veillonellales-Selenomonadales; f__Veillonellaceae; g__Veillonella
eb44896f88d	-0.0690188	0.11539836	0.55464099	0.69249974	d__Bacteria; p__Firmicutes; c__Clostridia; o__Lachnospirales; f__Lachnospiraceae; g__Stomatobaculum; s__uncultured_bacterium
X248d9c393f	0.01571409	0.02595279	0.54974364	0.69249974	d__Bacteria; p__Bacteroidota; c__Bacteroidia; o__Bacteroidales; f__Prevotellaceae; g__Prevotella
ae0c6cee8a6	-0.0759981	0.12839048	0.55868813	0.69354389	d__Bacteria; p__Firmicutes; c__Clostridia; o__Peptostreptococcales-Tissierellales; f__Anaerovoracaceae; g__[Eubacterium]_nodatum_group
e8cff5dd9314	0.04814484	0.08262964	0.56494508	0.69730364	d__Bacteria; p__Firmicutes; c__Bacilli; o__Lactobacillales
X29838c8386	0.04382179	0.07759721	0.57688615	0.70799664	s__uncultured_bacterium
X09251debfc	-0.0199217	0.03933715	0.6166936	0.7508476	d__Bacteria; p__Bacteroidota; c__Bacteroidia; o__Bacteroidales; f__Prevotellaceae; g__Prevotella; s__Prevotella_oulorum
X28a3212dff3	0.050314	0.10099282	0.62223019	0.7508476	d__Bacteria; p__Patescibacteria; c__Gracilibacteria; o__Absconditabacteriales_(SR1); f__Absconditabacteriales_(SR1); g__Absconditabacteriales_(SR1); s__SR1_bacterium

X47ad35356a	0.03894431	0.07828502	0.62211182	0.7508476	d__Bacteria; p__Firmicutes; c__Bacilli; o__Staphylococcales; f__Staphylococcaceae; g__Staphylococcus
X8e058482fc	0.01374004	0.03023484	0.65301576	0.78361891	d__Bacteria; p__Firmicutes; c__Clostridia; o__Lachnospirales; f__Lachnospiraceae; g__uncultured; s__Eubacterium_sp.
f7f2cea1e0c0	0.03106032	0.07023662	0.66176632	0.78973218	d__Bacteria; p__Firmicutes; c__Bacilli; o__Lactobacillales; f__Streptococcaceae; g__Streptococcus
cdce04d738e	0.04099884	0.09437092	0.66740342	0.79208318	d__Bacteria; p__Proteobacteria; c__Gammaproteobacteria; o__Pasteurellales; f__Pasteurellaceae; g__Aggregatibacter
c1b636001e0	-0.0275824	0.06540652	0.67643286	0.79686948	d__Bacteria; p__Firmicutes; c__Negativicutes; o__Veillonellales-Selenomonadales; f__Veillonellaceae; g__Veillonella
X6e086c4336	-0.023187	0.05543312	0.67881474	0.79686948	d__Bacteria; p__Firmicutes; c__Bacilli; o__Erysipelotrichales; f__Erysipelotrichaceae; g__Bulleidia; s__Bulleidia_extructa
e1dc096664c	-0.0238201	0.05925207	0.6907842	0.80653723	d__Bacteria; p__Actinobacteriota; c__Actinobacteria; o__Actinomycetales; f__Actinomycetaceae; g__Actinomyces; s__Actinomyces_dentalis
X83aed15ae1	-0.0221812	0.06431206	0.73288082	0.84203328	d__Bacteria; p__Actinobacteriota; c__Actinobacteria; o__Micrococcales; f__Micrococcaceae; g__Rothia
X400398fc36	0.00942495	0.02708947	0.7305796	0.84203328	d__Bacteria; p__Actinobacteriota; c__Actinobacteria; o__Bifidobacteriales; f__Bifidobacteriaceae; g__Alloscardovia; s__Alloscardovia_omnicolens
X3f6c45001fc	-0.0146328	0.04210526	0.73091064	0.84203328	d__Bacteria; p__Proteobacteria; c__Gammaproteobacteria; o__Burkholderiales; f__Neisseriaceae; g__Neisseria
X5310d435fd	0.01020073	0.03025665	0.73864974	0.84417113	d__Bacteria; p__Bacteroidota; c__Bacteroidia; o__Bacteroidales; f__Prevotellaceae; g__Prevotella; s__Prevotella_oulorum
f7c8e3b65c0	0.02689334	0.08644962	0.75812548	0.86186897	d__Bacteria; p__Proteobacteria; c__Gammaproteobacteria; o__Burkholderiales; f__Burkholderiaceae; g__Lautropia
X2737308c23	0.02261244	0.07737476	0.77226252	0.86761564	d__Bacteria; p__Actinobacteriota; c__Actinobacteria; o__Corynebacteriales; f__Corynebacteriaceae; g__Corynebacterium; s__Corynebacterium_durum
X185f287b8b	0.0233887	0.08050732	0.77269756	0.86761564	d__Bacteria; p__Patescibacteria; c__Saccharimonadia; o__Saccharimonadales; f__Saccharimonadaceae; g__TM7x; s__uncultured_bacterium
X9ead73ac54	0.04461057	0.15460567	0.77523064	0.86761564	s__Lautropia_sp.
e9fd215766b	0.02229981	0.08803488	0.80161904	0.89252429	d__Bacteria; p__Synergistota; c__Synergistia; o__Synergistales; f__Synergistaceae; g__Fretibacterium
X90b3d3969	-0.0162284	0.07616103	0.83287982	0.8995102	d__Bacteria; p__Firmicutes; c__Bacilli; o__Mycoplasmatales; f__Mycoplasmataceae; g__Mycoplasma
X37ff76f02e7	-0.0226005	0.1038222	0.82938066	0.8995102	d__Bacteria; p__Proteobacteria; c__Gammaproteobacteria; o__Pasteurellales; f__Pasteurellaceae; g__Haemophilus
X987a275a84	-0.0168569	0.07697285	0.82806323	0.8995102	d__Bacteria; p__Actinobacteriota; c__Actinobacteria; o__Actinomycetales; f__Actinomycetaceae; g__F0332; s__unidentified
ac5402de1dd	0.01720202	0.08064654	0.83259885	0.8995102	d__Bacteria; p__Bacteroidota; c__Bacteroidia; o__Bacteroidales; f__Bacteroidaceae; g__Bacteroides; s__Bacteroides_vulgatus
X46aaf5ecb5	0.02487658	0.11027086	0.82312695	0.8995102	d__Bacteria; p__Firmicutes; c__Clostridia; o__Lachnospirales; f__Lachnospiraceae; g__Oribacterium
X8b159e3b7b	0.01828122	0.08348749	0.82826465	0.8995102	d__Bacteria; p__Firmicutes; c__Clostridia; o__Peptostreptococcales-Tissierellales; f__Anaerovoracaceae; g__Mogibacterium
X2714c3e6dc	0.00927974	0.05157019	0.85855325	0.900434	d__Bacteria; p__Bacteroidota; c__Bacteroidia; o__Bacteroidales; f__Prevotellaceae; g__Alloprevotella
X4d861a31ac	-0.0091663	0.04608577	0.84382604	0.900434	d__Bacteria; p__Firmicutes; c__Clostridia; o__Peptostreptococcales-Tissierellales; f__Anaerovoracaceae; g__Family_XIII_UCG-001; s__Eubacterium_sp.
X5d62137a2	-0.0140368	0.07811036	0.85874724	0.900434	d__Bacteria; p__Firmicutes; c__Negativicutes; o__Veillonellales-Selenomonadales; f__Veillonellaceae; g__Dialister
X465c45bc4b	0.01577009	0.08398025	0.85183809	0.900434	d__Bacteria; p__Actinobacteriota; c__Actinobacteria; o__Actinomycetales; f__Actinomycetaceae; g__Actinomyces
a9497011c5b	0.02304641	0.11170071	0.83813123	0.900434	d__Bacteria; p__Spirochaetota; c__Spirochaetia; o__Spirochaetales; f__Spirochaetaceae; g__Treponema; s__Treponema_socranskii
X7892a2e5b5	-0.018034	0.10037961	0.85872814	0.900434	d__Bacteria; p__Bacteroidota; c__Bacteroidia; o__Bacteroidales; f__Prevotellaceae; g__Prevotella
dd3ff31d00fb	0.01210447	0.07055273	0.86500473	0.90261363	d__Bacteria; p__Spirochaetota; c__Spirochaetia; o__Spirochaetales; f__Spirochaetaceae; g__Treponema
X52b25decd3	0.00982124	0.08380059	0.90757189	0.9424785	d__Bacteria; p__Firmicutes; c__Negativicutes; o__Veillonellales-Selenomonadales; f__Selenomonadaceae; g__Selenomonas; s__Selenomonas_sp.
be4ccff9dd8d	0.00697162	0.06263976	0.9122041	0.94275639	d__Bacteria; p__Bacteroidota; c__Bacteroidia; o__Flavobacteriales; f__Flavobacteriaceae; g__Capnocytophaga
X9886d8901	0.00672411	0.07288475	0.92715149	0.94670547	d__Bacteria; p__Firmicutes; c__Bacilli; o__Lactobacillales; f__Aerococcaceae; g__Abiotrophia; s__uncultured_bacterium
X64cafef490d	0.00347158	0.03869574	0.92917389	0.94670547	d__Bacteria; p__Proteobacteria; c__Gammaproteobacteria; o__Burkholderiales; f__Neisseriaceae
c3e85f24357	0.00518924	0.05710958	0.92823376	0.94670547	d__Bacteria; p__Firmicutes; c__Clostridia; o__Lachnospirales; f__Lachnospiraceae; g__Blautia; s__Lachnospiraceae_bacterium
X15169180800	-0.0054769	0.08709444	0.95026542	0.96364944	d__Bacteria; p__Campilobacterota; c__Campylobacteria; o__Campylobacterales; f__Campylobacteraceae; g__Campylobacter
a6e07e769ad	0.00050244	0.01715331	0.97684076	0.98597012	d__Bacteria; p__Proteobacteria; c__Gammaproteobacteria; o__Burkholderiales; f__Neisseriaceae; g__Neisseria
faa2a7dbe2ac	0.00165347	0.08089728	0.98385346	0.98842952	d__Bacteria; p__Bacteroidota; c__Bacteroidia; o__Bacteroidales; f__Prevotellaceae; g__Prevotella; s__unidentified_eubacterium
a650f349bbd	0.00056609	0.04343511	0.98969414	0.98969414	d__Bacteria; p__Firmicutes; c__Bacilli; o__Lactobacillales; f__Streptococcaceae; g__Streptococcus

* positive coefficient = increased in preservative samples

Table S3. Significantly different ASVs by phylum

Phylum	Total Number of ASVs	Relative Abundace Analysis			Absolute Abundance Analysis		
		Non significant ASVs	Significant ASVs	Mean relative abundance	Non significant ASVs	Significant ASVs	Mean absolute abundance
**Firmicutes**	299	292	7	0.435	291	8	45987431
**Bacteroidetes**	238	225	13	0.188	223	15	26044675
**Proteobacteria**	103	102	1	0.231	101	2	18737910
**Actinobacteriota**	65	59	6	0.093	59	6	7989338
**Fusobacteria**	60	50	10	0.046	45	15	5723813
**Spirochete**	40	40	0	1.3E-03	40	0	42991
**Patescibacteria**	19	18	1	1.1E-03	19	0	176345
**Synergistota**	6	6	0	2.1E-04	6	0	6953
**Deinococcota**	5	5	0	7.5E-03	5	0	181
**Verrucomicrobia**	3	3	0	1.6E-03	3	0	14883
**Desulfobacterota**	2	2	0	4.3E-06	2	0	307
